# Modeling ErbB2-p130Cas interaction to design new potential anticancer agents

**DOI:** 10.1038/s41598-019-39510-w

**Published:** 2019-02-28

**Authors:** Andrea Costamagna, Matteo Rossi Sebastiano, Dora Natalini, Matilde Simoni, Giorgio Valabrega, Paola Defilippi, Sonja Visentin, Giuseppe Ermondi, Emilia Turco, Giulia Caron, Sara Cabodi

**Affiliations:** 1 Department of Molecular Biotechnology and Health Sciences, University of Torino, Via Nizza 52, 10126 Torino, Italy; 2 Institut für Pharmacologie, Universität Bern, Inelspital INO-F, CH-3010 Bern, Switzerland; 3 Candiolo Cancer Institute, FPO-IRCCS-Candiolo, (TO), 10060 Italy

**Keywords:** Target validation, Medicinal chemistry

## Abstract

The ErbB2 receptor tyrosine kinase is overexpressed in approximately 15–20% of breast tumors and associated with aggressive disease and poor clinical outcome. p130Cas represents a nodal scaffold protein regulating cell survival, migration and proliferation in normal and pathological contexts. p130Cas overexpression in ErbB2 human breast cancer correlates with poor prognosis and metastasis formation. Recent data indicate that p130Cas association to ErbB2 protects ErbB2 from degradation, thus enhancing tumorigenesis. Therefore, inhibiting p130Cas/ErbB2 interaction might represent a new therapeutic strategy to target breast cancer. Here we demonstrate by performing Molecular Modeling, Molecular Dynamics, dot blot, ELISA and fluorescence quenching experiments, that p130Cas binds directly to ErbB2. Then, by structure-based virtual screening, we identified two potential inhibitors of p130Cas/ErbB2 interaction. Their experimental validation was performed *in vitro* and in ErbB2-positive breast cancer cellular models. The results highlight that both compounds interfere with p130Cas/ErbB2 binding and significantly affect cell proliferation and sensitivity to Trastuzumab. Overall, this study identifies p130Cas/ErbB2 complex as a potential breast cancer target revealing new therapeutic perspectives for protein-protein interaction (PPI).

## Introduction

Breast cancer is the second most common cancer worldwide after lung cancer, the fifth most common cause of cancer death, and the leading cause of cancer death in women^1^.

In the last decades breast cancer treatment has greatly improved due to the development of targeted therapies against Receptor Tyrosine Kinases (RTKs) whose hyperactivation or overexpression leads to increased cell proliferation, survival and transformation^2^. In particular, there is a huge interest among medicinal chemists for inhibitors of the epidermal growth factor receptor (EGFR) family which includes four structurally related receptor tyrosine kinases (ErbB1-4). The ErbB proteins function as homo- and hetero- dimerizer upon ligand binding, but ErbB2 is an orphan receptor and its activation upon homo- and hetero- dimerization is ligand-independent. Notably, over-expression or amplification of ErbB2 tyrosine kinase occurs in up to 20% of human breast cancers, where it is predictive of aggressive disease and poor clinical outcome^3^. Several anti-ErbB2 therapies have been recently proposed including receptor tyrosine kinase inhibitors and humanized monoclonal antibodies. Among them, Trastuzumab was approved for the treatment of breast cancers overexpressing ErbB2, alone or in combination with standard chemotherapy. The mechanism of action of Trastuzumab is complex and not well understood, resulting in modest downregulation of the ErbB2 receptor^4,5^.

p130Cas is an adaptor protein devoid of any enzymatic or transcriptional activity and its modular structure with various binding motifs allows the formation of multi-protein signaling complexes. This results in the induction and/or maintenance of signaling pathways with pleiotropic effects on cell motility, cell adhesion, cytoskeleton remodeling, invasion, survival and proliferation. The relevance of p130Cas adaptor protein in cancer has been extensively supported. p130Cas overexpression has been detected in human breast, prostate, ovarian, lung, colorectal, pancreatic and hepatocellular carcinoma, as well as in glioma, melanoma, anaplastic large cell lymphoma and chronic myelogenous leukemia, although the exact mechanisms that drive p130Cas overexpression in cancer have not yet been identified^6–8^.

Specifically, in ErbB2-positive breast cancer, p130Cas overexpression correlates with poor prognosis and increase metastatization^9^. From a molecular point of view, it has been demonstrated that p130Cas is a crucial component of a functional molecular complex consisting in ErbB2, c-Src, and FAK supporting cell proliferation and invasion^10^. Recently, it was reported that p130Cas is able to stabilize ErbB2, by binding and protecting it from autophagic degradation. Indeed, p130Cas binding to ErbB2 does not allow the association of CHIP and Cbl, the major E3 ubiquitin ligases binding ErbB2, possibly through steric hindrance^5^.

These data also indicate that high levels of p130Cas expression inversely correlate with ErbB2 sensitivity to Trastuzumab. The mechanism through which p130Cas mediates resistance to Trastuzumab might rely on the increased ErbB2 stability to the cell membrane. This increased stabilization of ErbB2 by p130Cas might be the crucial event driving breast cancer progression and resistance, strengthening the relevance of p130Cas as putative therapeutic target to overcome resistance to Trastuzumab^5^.

In addition, alternative ErbB2 targeted therapies, such as Pertuzumab or Trastuzumab-Emtansine (T-DM1) were developed. Combination of Trastuzumab and Pertuzumab treatment have shown a statistically significant increase in overall survival of ErbB2 positive metastatic breast cancer patients^11,12^, while T-DM1 improved overall survival of ErbB2-positive metastatic patients that failed other ErbB2-targeted drugs^13^.

Even though these therapies extend overall survival in responders, a high percentage of patients develop resistance. Indeed, Trastuzumab/Pertuzumab/Docetaxel versus Trastuzumab/Docetaxel combination treatment increased the patients’ responsiveness from 29% to 45.8%^14^, while T-DM1 failed to induce response in 57% of patients^15^.

Therefore, alternative strategies are needed. Tyrosine kinase inhibitors (TKI) have been proposed as alternative treatments to counteract Trastuzumab resistance, but they have some limitations. The most critical drawback is the development of drug resistance due to secondary mutations of the targeted receptors or to compensatory activation of other RTKs that renders the therapies ineffective.

Consequently, key protein-protein interactions involved in RTKs signaling could represent a major source of novel pharmacological targets. In contrast to enzyme inhibitors (es. TKI), protein-protein interaction inhibitors target larger contact surface often involving more than two contact pockets composed of multiple amino acids located in different region of the protein^16,17^. This in turn, should limit or delay development of drug resistance due to secondary mutation of the targeted receptor.

Taken together this evidence suggests important therapeutic and translational applications of p130Cas in ErbB2 breast cancer and supports the hypothesis that p130Cas/ErbB2 interaction can serve as a potential target for the discovery and development of new anticancer agents, that can be used in combination with standard therapy to manage and control Trastuzumab resistance.

To verify this hypothesis, we have initially used computer strategies, *in vitro* proteomic approach and cellular models and showed that the SH3 domain of p130Cas binds a specific sequence of ErbB2 intracellular domain. Then, a structure-based virtual screening (SBVS) procedure identified molecules with potential inhibitory activity *vs* p130Cas/ErbB2 interaction. Two *ad hoc* selected hits resulting from the computational screening were experimentally tested both *in vitro* and in breast cancer cell lines. Finally, the physico-chemical and ADME-Tox profiles of the two molecules were predicted to exploit their full potential as drugs.

Overall this study supports p130Cas/ErbB2 complex as a potential breast cancer target and shows the druggability of this protein-protein interaction (PPI) that might benefit of a more advanced optimization effort for therapeutic applications.

## Results

### Modeling the interaction of p130Cas and ErbB2

Literature analysis suggests that p130Cas scaffold might independently associate with ErbB2 in a direct way. Due to the structural complexity of the partners, the study focused only on the interaction region between the two proteins. Therefore, we searched for protein-protein binding sequences on ErbB2 cytosolic portion by the online algorithm Eukaryotic Linear Motif (ELM) (www.elm.eu). ELM is a bioinformatic resource combining experimental evidences with a predictive algorithm that returns the biological function (experimentally determined if possible or predicted) of recognized short sequences in eukaryotic proteins^18^.

**Figure 1:**
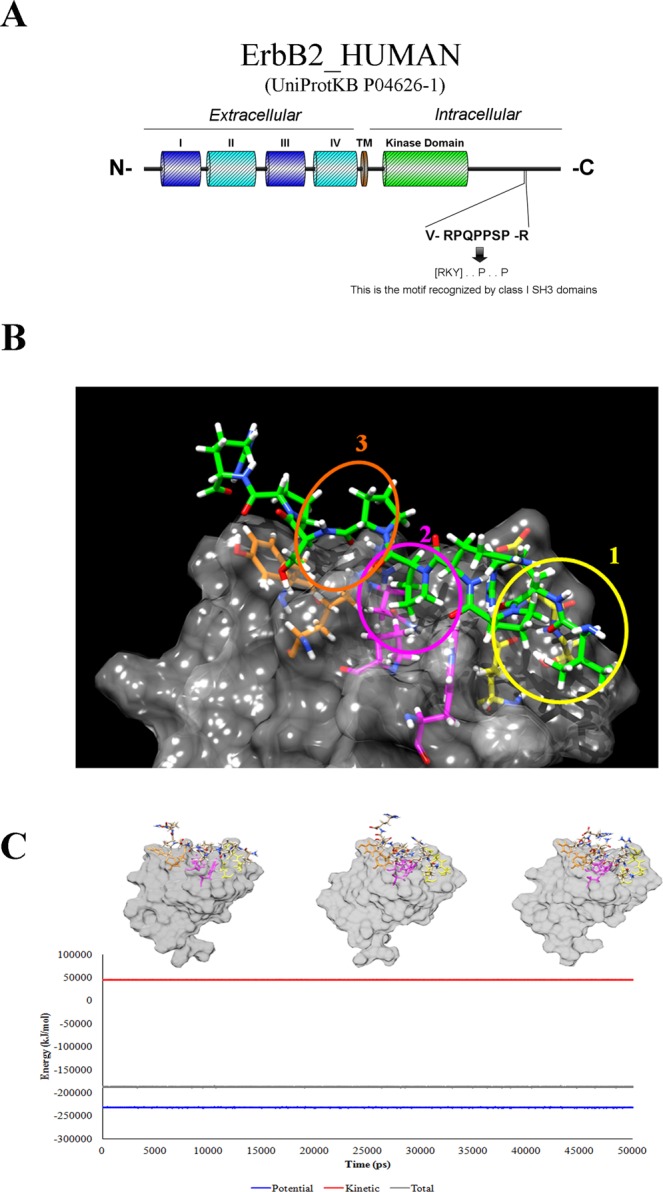
Modeling of SH3_p130Cas/PPII_ErbB2 interaction. (**A**) Schematic representation of the ErbB2 receptor. The class I SH3 ligand in position 1146–1152 shown in the inset is located in the unstructured carboxy-terminal portion of ErbB2 receptor. (**B**) PPII_**ErbB2** peptide is shown in green, hydrophobic and positive charge interaction surfaces are in grey, negative charge interaction surfaces in cyan. The three interaction sites (1, 2 and 3) are circled in white. The PPII_**ErbB2** Arg2, Pro5 and Pro8 side-chains are highlighted in green. Interacting residues for SH3_**p130Cas** are described in the text. (**C**) Not standard conditions stages (black dots), standard condition stages (light blue dots), U_ab_ trend line (green), SH3_**p130Cas** (blue chain), PPII_**ErbB2** (red chain). This is shown by the four snapshots in Fig. 2 that represent four dynamics stages distributed along the simulation (standard conditions P [90;110] kPa and T [230;310] K). Stage 2 represents a “peak” in the trend of U_ab_ values; it has been chosen to demonstrate that this U_ab_ variation does not lead to the dissociation of the complex. Apart from stage 2 (and stage 0, the starting point from SH3_**p130Cas**/PPII_**ErbB2** interaction model) no substantial variation in the U_ab_ general trend values were observed, suggesting that there is no difference in complex stability between each couple of stages.

The ELM sequence analysis of C-terminal cytosolic domain of ErbB2 predicts the presence in position 1145–1153 of a -V[RPQPPSP]R- nine amino acid sequence (PPII_**ErbB2**). The motif is a polyproline type II domain, i.e. a left hand, trans proline coil whose 1-4-7 residue-side chains have the same spatial orientation (RxxPxxP) and could act as a binding site for the SH3 domain of p130Cas (Fig. 1A)^19–21^. On the basis of these evidences, an *in-silico* interaction model of the PPII_**ErbB2** peptide and p130Cas SH3 domain (SH3_**p130Cas**) was thus built using a template-based modeling strategy^22^. The structure of the SH3 domain of p130Cas was downloaded from the PDB (PDB code 1WYX, resolution = 1.1 Å)^23^. PPII_**ErbB2** was modeled using the crystallographic structure of the complex between PD1R, a synthetic peptide with polyproline type II conformation, and the SH3 domain of p85 subunit of PI3K (highly homologous to SH3_**p130Cas**) (PDB code 3I5R, resolution 1.7 Å). The interaction model (see Experimental Section) shows three contact regions (named 1, 2 and 3 in Fig. 1B): the first concerns PPII_**ErbB2** Arg2 that interacts with SH3_**p130Cas** Glu15 and Glu19 and forms a network of reinforced hydrogen bonds, the second and the third are hydrophobic interactions involving Pro5 and Pro8 (PPII_**ErbB2**) and apolar pockets of SH3_**p130Cas**.

### Molecular Dynamics (MD) simulations

MD simulations were performed to check the stability over the time of the interaction described above. Results are resumed in Fig. 1C which shows the energy variation with the time (t). No dissociation of the SH3_**p130Cas**/PPII_**ErbB2** complex is observed. As a negative control we mutated the three PPII residues mainly involved in the interaction into amino acids less prone to stabilize the binding (positive charged Arg2 into negative charged Glu, small Pro5 and Pro8 into the larger Asn) and applied the same MD protocol. Results show a weaker interaction between the mutated peptide and SH3_**p130Cas** (see Fig. S1 in Supporting Information).

Furthermore, MD simulations were also successfully used to support the binding orientation, – VRPQPPSPR – of the SH3 domain to the polyproline domain (see Fig. S2 in Supporting Information).

### *In vitro* validation of the direct interaction between SH3 domain of p130Cas and ErbB2

We expressed and purified recombinant proteins for p130Cas and ErbB2 as described in the Experimental Section and Table 1 (see Fig. S3 in Supporting Information). Three domains of p130Cas, i.e. the SH3 domain (SH3), the substrate domain (YXXP) and the carboxy-terminal domain (CT), were tested for their ability to bind two recombinant proteins for the C-terminal tail of ErbB2 (*MBP*_shortPPII_**ErbB2** and *MBP*_longPPII_**ErbB2**, see Table 1), containing the putative polyproline type II sequence, in two different *in vitro* assays.

**Table 1:** List of constructs used.

ErbB2	
PPII_**ErbB2**	Polyproline domain type II identified in ErbB2 protein sequence by ELM algorithm. Sequence: V[RPQPPSP]R Position: 1145–1153 in the ErbB2 protein sequence (Isoform 1, identifier: P04626-1).
*MBP*_longPPII_**ErbB2**	Recombinant protein containing an N-terminal *MBP* tag followed by a fragment of ErbB2 cytosolic portion (aminoacids 1103–1255). This fragment starts 30 aminoacids before the putative polyproline domain (PPII_**ErbB2**) and ends 30aa after.
*MBP*_shortPPII_**ErbB2**	Recombinant protein containing an N-terminal *MBP* tag followed by a fragment of ErbB2 cytosolic portion (aminoacids 1103–1194). This fragment starts 30 aminoacids before the putative polyproline domain (PPII_**ErbB2**) and ends with the endogenous C-terminal of the protein.
*MBP*_ctrl	*MBP* tag (Maltose Binding Protein from E. Coli) used as affinity purification tag for ErbB2 recombinant constructs. MPB protein alone was used as control.
*MBP*_R → D_shortPPII_**ErbB2**	This recombinant protein was generated from *MBP*_shortPPII_**ErbB2** by site-directed mutagenesis. It carries an Arginine to Aspartate (R to D) substitution at position 44 (position 1146 in the original ErbB2 sequence). This substitution alters the putative polyproline domain identified in ErbB2 protein sequence from V[**R**PQPPSP]R to V[**D**PQPPSP]R.
*MBP*_PP → AA_shortPPII_**ErbB2**	This recombinant protein was generated from *MBP*_shortPPII_**ErbB2** by site-directed mutagenesis. It carries two Proline to Alanine (P to A) substitutions at position 47 and 50 (position 1149 and 1152 in the original ErbB2 sequence). These substitutions alter the putative polyproline domain identified in ErbB2 protein sequence from V[RPQ**P**PS**P**]R to V[RPQ**A**PS**A**]R.
*MBP*_RPP → DAA_shortPPII_**ErbB2**	This recombinant protein was generated from *MBP*_shortPPII_**ErbB2** by site-directed mutagenesis. It carries an Arginine to Aspartate (R to D) substitution at position 44 (position 1146 in the original ErbB2 sequence) and two Proline to Alanine (P to A) substitutions at position 47 and 50 (position 1149 and 1152 in the original ErbB2 sequence). These substitutions alter the putative polyproline domain identified in ErbB2 protein sequence from V[**R**PQ**P**PS**P**]R to V[**D**PQ**A**PS**A**]R.
*MBP*_deleter_shortPPII_**ErbB2**	This recombinant protein was generated from *MBP*_shortPPII_**ErbB2** by site-directed mutagenesis. This protein carries a 7-aminoacid deletion from position 44 to 50 (position 1146–1152 in the original ErbB2 sequence). This deletion abrogates the putative polyproline domain identified in ErbB2 protein sequence.
**p130Cas**	
*GST*_SH3_**p130Cas**	Recombinant protein containing an N-terminal *GST* tag followed by a fragment of p130Cas protein (aminoacids 3–71). This fragment represents the SH3 domain of p130Cas as described in^23^.
*GST*_YXXP_**p130Cas**	Recombinant protein containing an N-terminal *GST* tag followed by a fragment of p130Cas protein (aminoacids 80–400). This fragment represents a large central substrate domain composed of 15 repeats of a four amino acid sequence (YXXP)^23^.
*GST*_CT_**p130Cas**	Recombinant protein containing an N-terminal *GST* tag followed by a fragment of p130Cas protein (aminoacids 654–874). This fragment represents the carboxy-terminal (CT) domain of p130Cas which possess consensus binding sites for the SH2 and SH3 domains of Src^23^.
*GST*_ctrl	*GST* tag (Glutathione S-Transferase from Schistosoma japonicum) used as affinity purification tag for p130Cas recombinant constructs. *GST* protein alone was used as control.

**Figure 2:**
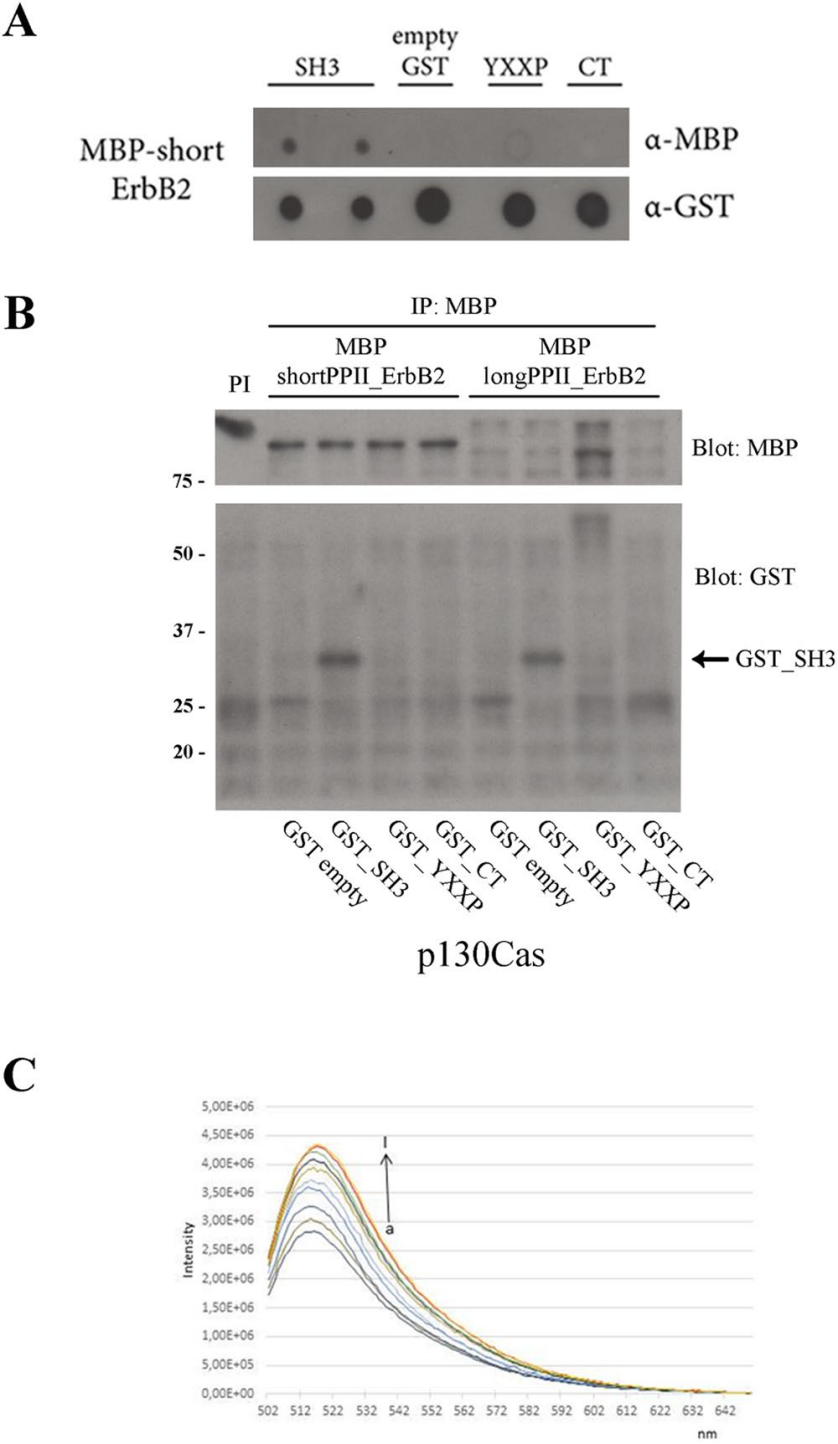
SH3_**p130Cas** directly interacts with shortPPII_**ErbB2** and longPPII_**ErbB2** *in vitro*. (**A**) Dot blot experiment revealed a direct interaction between *GST*_SH3_**p130Cas** and *MBP*_shortPPII_**ErbB2** (lane 1–2), using anti-*MBP* antibody. No interactions were detected with control *GST*_ctrl, *GST*_YXXP_**p130Cas** and *GST*_CT_**p130Cas** recombinant proteins (lane 3–5). Normalization was performed with anti-*GST* antibody. (**B**) *GST*_SH3_**p130Cas** was able to interact with *MBP*_shortPPII_**ErbB2** and *MBP*_longPPII_**ErbB2** in solution (lane 2 and 6). After standard immunoprecipitation with anti-*MBP* antibody, the interaction was revealed with anti-*GST* antibody. No interaction or little aspecific interaction were detected with control *GST*_ctrl (lane 1 and 5), *GST*_YXXP_**p130Cas** (lane 3 and 7) and *GST*_CT_**p130Cas** (lane 4 and 8) recombinant proteins. (**C**) Enhancement fluorescence of Fl-*GST*_SH3_**p130Cas** (3.0 μM) in presence of increasing concentrations of *MBP*_shortPPII_**ErbB2** (0,05, 0.1, 0.3,0.75, 1, 2, 3 µM).

As shown in Fig. 2A, dot blot assays were performed by spotting the *GST*-tagged **p130Cas** domains on nitrocellulose membrane and probing them with *MBP*_shortPPII_**ErbB2**. Western blot analysis clearly revealed that the interaction between SH3_**p130Cas** and shortPPII_**ErbB2** was specific as no binding was observed with other p130Cas domains (Fig. 2A).

As a further validation of the SH3_**p130Cas**/PPII_**ErbB2** interaction, *in vitro* binding assays were performed. *GST*- and *MBP*- tagged recombinant proteins were kept in solution overnight at 4 °C and then immunoprecipitated with anti-*MBP* antibody. Western blot analysis indicates that the interaction between p130Cas and ErbB2 indeed occurred through the SH3 domain of p130Cas (Fig. 2B).

To quantify the entity of the interaction, we performed a fluorescence assay. As shown in Fig. 2C an increasing concentration of *MBP*_shortPPII_**ErbB2** causes an enhancement in fluorescence intensity emission of Fl-*GST*_SH3_**p130Cas**. Fluorescence data were analyzed with a nonlinear least-squares fit (Fig. S4a) and produced a Kd = 4.07 × 10^−8^ M, a value that could be ascribed to a moderate-strength interaction.

Overall *in vitro* assays demonstrated that p130Cas can effectively bind ErbB2 receptor in a direct way, and that this association is mediated by the SH3 domain of p130Cas.

### Validation of key aminoacids involved in p130Cas/ErbB2 interaction

**Figure 3:**
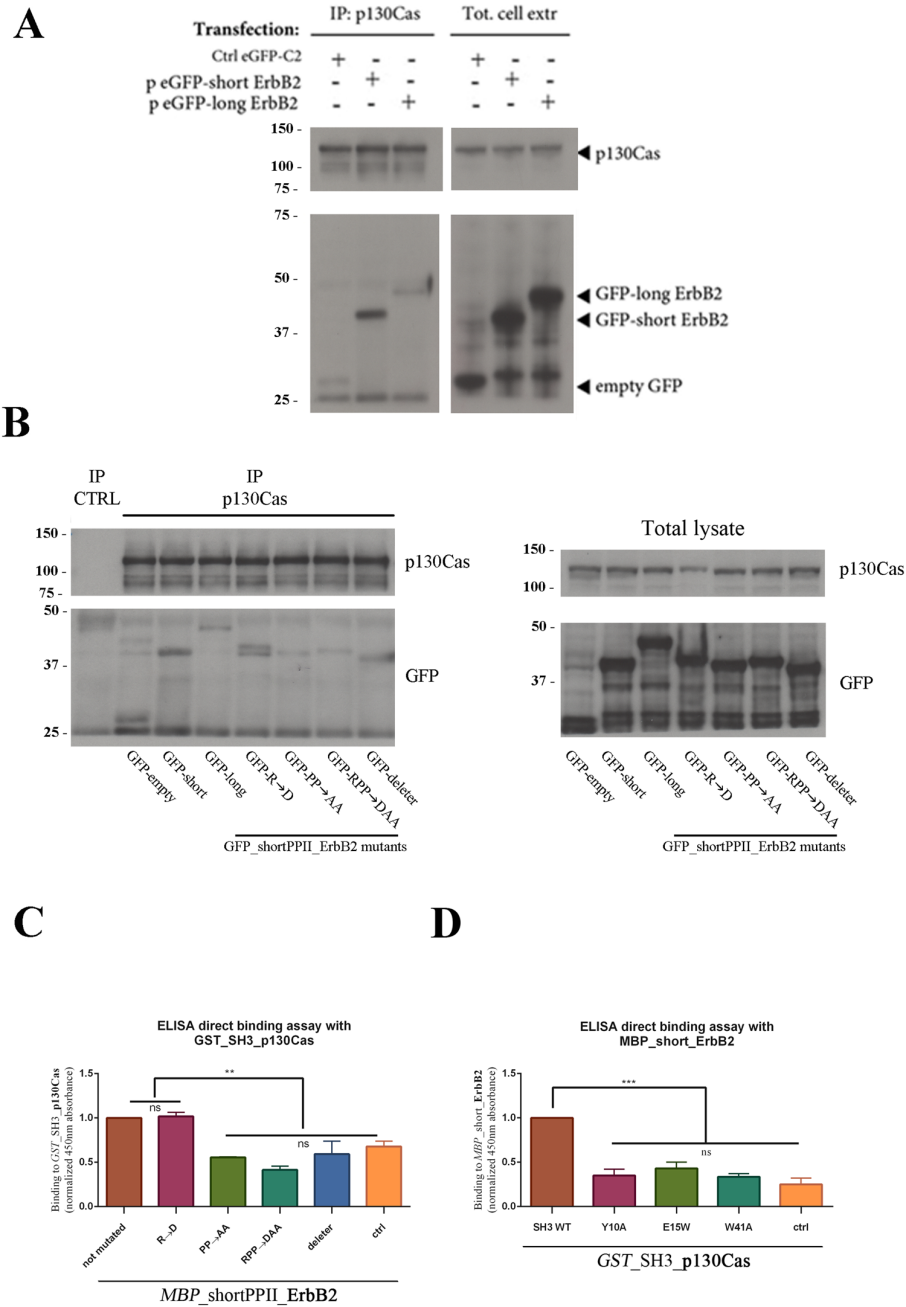
Validation of p130Cas/ErbB2 direct interaction in 293T cell line. (**A**) 293T cells were transfected with GFP-tagged constructs for shortPPII_**ErbB2** and longPPII_**ErbB2**. Five days after transfection, endogenous p130Cas was immunoprecipitated with anti-p130Cas antibody. Interacting proteins were detected with anti-*GFP* antibody. Endogenous p130Cas interacts with both *GFP*_shortPPII_**ErbB2** and *GFP*_longPPII_**ErbB2**, although little aspecific interaction was detected with control *GFP*_ctrl (left). Total cell extracts were blotted with anti-p130Cas and anti-*GFP* antibodies to assess transfection efficiency (right). (**B**) 293T cells were transfected with mutant forms of *GFP*_shortPPII_**ErbB2**. Five days after transfection, endogenous p130Cas was immunoprecipitated with anti-p130Cas antibody. Interactions were revealed using anti-*GFP* antibody (left). Mutation of the cationic residue Arg2 slightly reduced *GFP*_shortPPII_**ErbB2** binding to endogenous p130Cas; conversely, mutations affecting Pro5 and Pro8 markedly decreased their association, as well as deletion of the PPII motif. Total cell extracts were blotted with anti-p130Cas and anti-*GFP* antibodies to assess mutants expression (right). (**C**) ELISA direct binding assay recapitulated results obtained using Co-Immunoprecipitation experiments in 293T cell line. Mutation of the cationic residue Arg2 does not affect the binding of *MBP*_shortPPII_**ErbB2** to *GST*_SH3_**p130Cas**; conversely, mutations affecting Pro5 and Pro8 markedly decreased their association, as well as deletion of the PPII motif. Data are represented as mean ± SD. **p < 0.01. (**D**) ELISA direct binding assay was performed with *GST*_SH3_**p130Cas** mutants to assess their binding to *MBP*_shortPPII_**ErbB2**. Recombinant SH3 domains carrying mutations in the three main interaction sites show impaired binding with PPII-containing recombinant protein, validating the importance of these residues for the interaction between SH3 domain and polyproline peptides. Data are represented as mean ± SD. ***p < 0.001.

To avoid false positive results due to the incorrect folding of proteins expressed in bacteria, we validated the p130Cas/ErbB2 interaction in 293T mammalian cell line that express normal levels of p130Cas but no detectable expression levels of ErbB2. We cloned and transfected plasmids containing *GFP*-tagged version of ErbB2 recombinant proteins (*GFP*_shortPPII_**ErbB2** and *GFP*_longPPII_**ErbB2**) as described in the Experimental Section. Five days after transfection, standard co-immunoprecipitation was performed using anti-p130Cas mouse antibody. *GFP*-tagged ErbB2 constructs co-immunoprecipitated with endogenous p130Cas in 293T cell line and thus false positive results due to the misfolding of recombinant proteins were excluded (Fig. 3A).

To verify in a cellular model that the interaction between p130Cas and ErbB2 occurs between p130Cas SH3 domain and the PPII domain of ErbB2, mutagenesis experiments were performed by modifying key residues of the polyproline type II motif of ErbB2. Specifically, cationic Arg2 was mutated to the anionic residue Asp; Pro5 and Pro8 were mutated to Ala. Proline is unique among the 20 common amino acids in having its side chain cyclized onto the backbone nitrogen atom, conferring it special chemical and physical characteristics that cannot be mimic by other amino acids^24^. Pro-Ala substitution was chosen to at least partially maintain the little size and the hydrophobic interactions of proline residue. We also generated a mutant in which the PPII domain of ErbB2 was completely deleted (named “deleter”) to assess if this motif was essential to drive p130Cas/ErbB2 interaction. Four different mutants for *GFP*_shortPPII_**ErbB2** were generated as described in the Experimental Section to perform co-immunoprecipitation in 293T cell line (Fig. 3B). The same mutants were also produced as *MBP*-tagged recombinant proteins to perform an *in vitro* ELISA direct binding assay (Fig. 3C).

Both experiments show that mutagenesis of key residues in PPII_**ErbB2** sequence alters its binding to SH3 domain of p130Cas. Interestingly, the substitution of Pro5 and Pro8 to Ala strongly affects the amount of binding, while the substitution of Arg to Asp does not impair the binding suggesting that the reinforced hydrogen bond plays a minor role in the interaction. On the contrary, mutation and/or deletion of the core PxxP motif (either by Proline substitution or by complete deletion of the PPII motif) strongly reduced SH3_**p130Cas**/shortPPII_**ErbB2** association, *in vitro* and in cellular model, demonstrating the importance of PPII located residues as suggested by the in-silico molecular model.

The same strategy was used to validate the PPII-binding pockets in the SH3 domain of p130Cas. Three SH3-mutants carrying mutations in the three main sites of the interaction were generated and subjected to an *in vitro* ELISA direct binding assay to evaluate their ability to bind ErbB2 recombinant proteins (Fig. 3D). Aromatic residues were changed to alanine to replace bulky hydrophobic residues with small apolar residues (Y10A and W41A). In addition, a conserved hydrophilic residue was substituted with a large hydrophobic residue (E15W). These mutations involved aminoacids exposed to the solvent on the surface of the SH3 domain and are not implicated in any contacts to main-chain atoms that maintain the structural stability of the SH3 domain^25^. Notably, all SH3 mutants failed to associate with the PPII domain of ErbB2 (Fig. 3D).

### Structure-based virtual screening (SBVS)

Once validated, the molecular model for p130Cas/ErbB2 interaction was used to run a SBVS of the ZINC database using FLAP software (see Experimental Section). The rational was to find candidates that bind the SH3 domain of p130Cas (SH3_**p130Cas**) using the same interaction pockets used by polyproline ErbB2 sequence (PPII_**ErbB2**) (see above) thereby inhibiting the binding of ErbB2 to p130Cas.

Screening results are in Table S2 (Pocket Point Radius = 2 Å), which shows the ten molecules expected to have the best capacity to inhibit the SH3_**p130Cas**/PPII_**ErbB2** interaction ranked by their Glob-Prod index (see Experimental Section). Potential hits from this SBVS were screening for PAINS using the tool implemented in ZINC15 (http://zinc15.docking.org/patterns/home/) to exclude the presence of known classes of assay interference groups in **1** and **2**.

**Figure 4:**
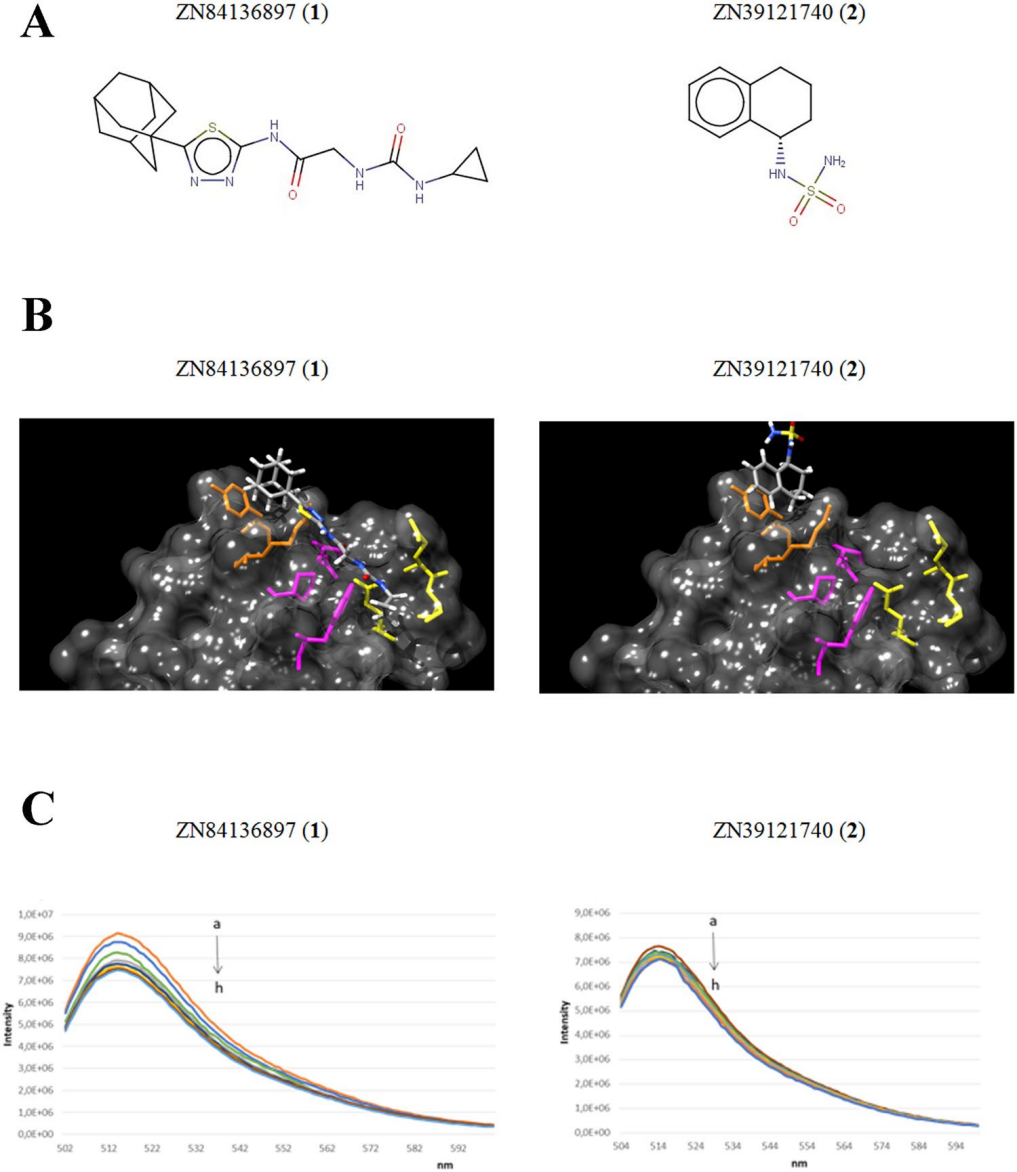
Structure-based virtual screening identifies compounds with potential inhibitory properties. (**A**) Chemical structures of two high ranked compounds from the SBVS: ZINC84136897 (**1**) and ZINC39121740 (**2**). (**B**) Binding mode of **1** (left panel) and **2** (right panel) with SH3_**p130Cas**. The selected compounds showed different binding modalities since **1** exploits its action by interacting with SH3_**p130Cas** in three regions, whereas the interaction of **2** with SH3_**p130Cas** is driven by hydrogen bonding (HB) interactions. (**C**) Fluorescence quenching spectra of the complex Fl-*GST*_SH3_**p130Cas** (0.07 μM) in the absence and presence of **1** (0, 0.07, 0.21, 0.7, 3.5, 7.0, 14, 21 μM) (left panel) and in the absence and presence of **2** (0, 2.1, 3.5, 5.5, 7.0, 10, 15, 21 μM) (right panel) demonstrating the binding between the selected compounds and the target protein.

ZINC84136897 (**1**) and ZINC39121740 (**2**) (chemical structures in Fig. 4A) were selected to submit to experimental validation. Compound **1** is larger than **2** (MW = 375) and could be described as a urea derivative. Compound **2** is a relatively small compound (MW = 226) bearing a sulfonamide group. The choice of these two compounds also allows to explore the impact on the inhibitory activity of different binding modes since **1** exploits its action by interacting with p130Cas in three regions, whereas the interaction of **2** with p130Cas is driven by hydrogen bonding (HB) interactions (Fig. 4B).

### *In vitro* validation of the compounds identified by virtual screening

To experimentally validate the binding of compound **1** and **2** on *GST*_SH3_**p130Cas** recombinant protein, fluorescence quenching experiments were performed.

The fluorescence spectra of Fl-*GST*_SH3_**p130Cas** in the absence and in the presence of **1** and **2** at different concentrations are shown in Fig. 4C. Fl-*GST*_SH3_**p130Cas** shows a strong fluorescence emission at 518 nm and its fluorescence intensity decreases gradually with the increase of compound’s concentration. The analysis of equilibrium dissociation constants showed values with different order of magnitude, K_D_ = 2.0 × 10^−7^ M for compound **1** and K_D_ = 5.0 × 10^−6^ M for compound **2** (Fig. 4C left and right panel, respectively) (see also Fig. S4b,c).

**Figure 5:**
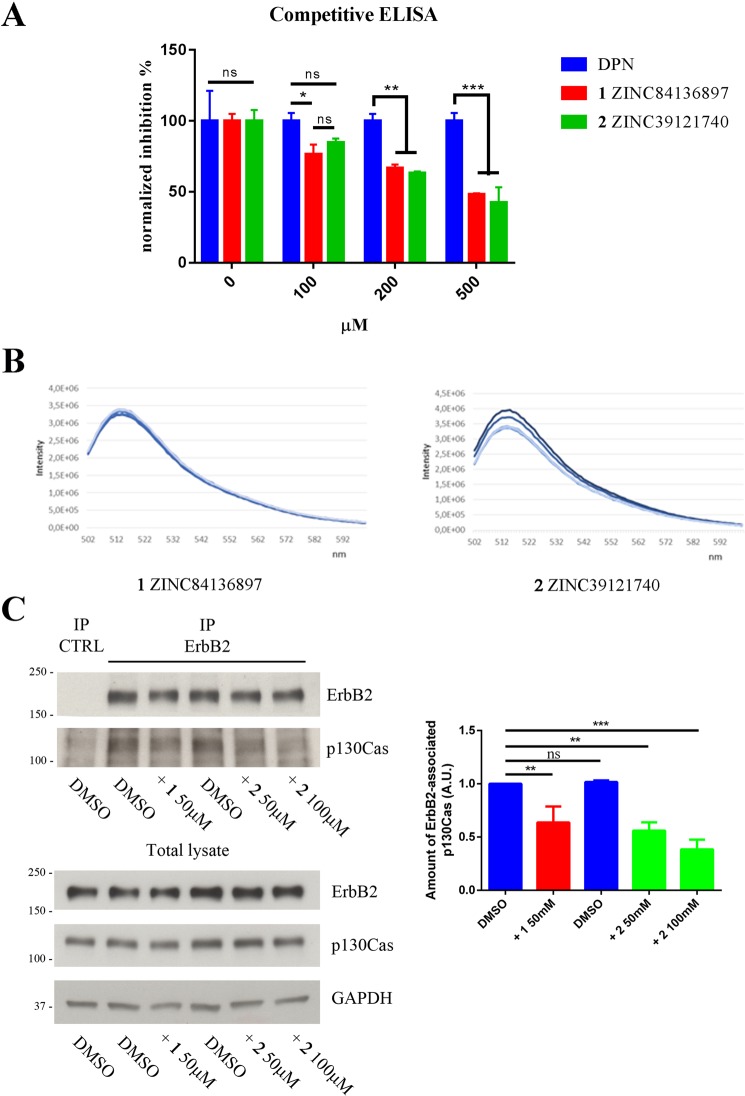
Screened compounds inhibit *GST*_SH3_**p130Cas**/*MBP*_shortPPII_**ErbB2** association *in vitro*. (**A**) Competitive ELISA binding assay was performed to validate two high ranked selected compounds (**1** and **2**) as inhibitor of p130Cas/ErbB2 interaction. Both compounds demonstrated dose-dependent inhibition of the association between *GST*_SH3_**p130Cas**/*MBP*_shortPPII_**ErbB2** purified recombinant proteins *in vitro*. DPN (Diarylpropionitrile) was low-ranked by virtual screening and therefore used as control for non-specific inhibition (see Experimental Section). (**B**) Fluorescence spectra of the complex Fl-*GST*_SH3_**p130Cas**/**1** (0.01 μM and 3 μM respectively) in presence of increasing concentrations of *MBP*_shortPPII_**ErbB2** (0.1, 0.3, 0.5, 0.75 μM (left panel); the complex Fl-*GST*_SH3_**p130Cas**/**2** (0.01 μM and 1 μM respectively) in presence of increasing concentrations of *MBP*_shortPPII_**ErbB2** (0.1, 0.3, 0.5, 0.75μM (right panel). (**C**) Co-immunoprecipitation experiment in BT474 cell line. Cells were pre-treated with selected inhibitory compounds for 24 hours and subjected to immunoprecipitation with anti-ErbB2 antibodies. Western blot with anti-p130Cas antibodies revealed that both compounds were able to reduce the amount of endogenous p130Cas/ErbB2 complex in BT474 cells (upper left panel) and quantified by densitometric analysis (right panel). Total cell extracts blotted with anti-ErbB2 and anti-p130Cas revealed no changes in ErbB2 or p130Cas expression during the treatment (lower left panel). GAPDH expression was used as loading control. Data are represented as mean ± SD. **p < 0.01, ***p < 0.001.

The two compounds were then tested *in vitro* using a competitive ELISA binding assay, which demonstrated their inhibitory activity against the interaction between *GST*_SH3_**p130Cas** and *MBP*_shortPPII_**ErbB2** recombinant proteins (Fig. 5A). The assay nicely showed reduced interaction between p130Cas and ErbB2 recombinant proteins with increasing concentration of tested compounds, while no modulation was revealed with increasing concentration of DPN (non-specific control). This experimental evidence validates the SH3 domain of p130Cas as possible pharmacological target since its binding to the polyproline domain of ErbB2 is amenable for modulation by inhibitory compounds **1** and **2**.

As a further confirmation of the **1** and **2** efficacies of inhibiting *GST*_SH3_**p130Cas**/*MBP*_shortPPII_**ErbB2** interaction, fluorescence quenching experiments were performed. As shown in Fig. 5B, compound **1** is more efficient in preventing the binding between Fl-*GST*_SH3_**p130Cas** and *MBP*_shortPPII_**ErbB2**, compared to compound **2** (Fig. 5B left and right panel, respectively).

To assess whether these compounds could interfere with p130Cas/ErbB2 association in a cellular context, ErbB2-positive BT474 breast cancer cells were pre-treated for 24 hours with **1** or **2** and subjected to co-immunoprecipitation experiments. As shown in Fig. 5C, treatment with selected inhibitory compounds reduced the association of p130Cas to ErbB2 also in BT474 breast cancer cells.

### Validation in cellular models of the identified inhibitors

**Figure 6:**
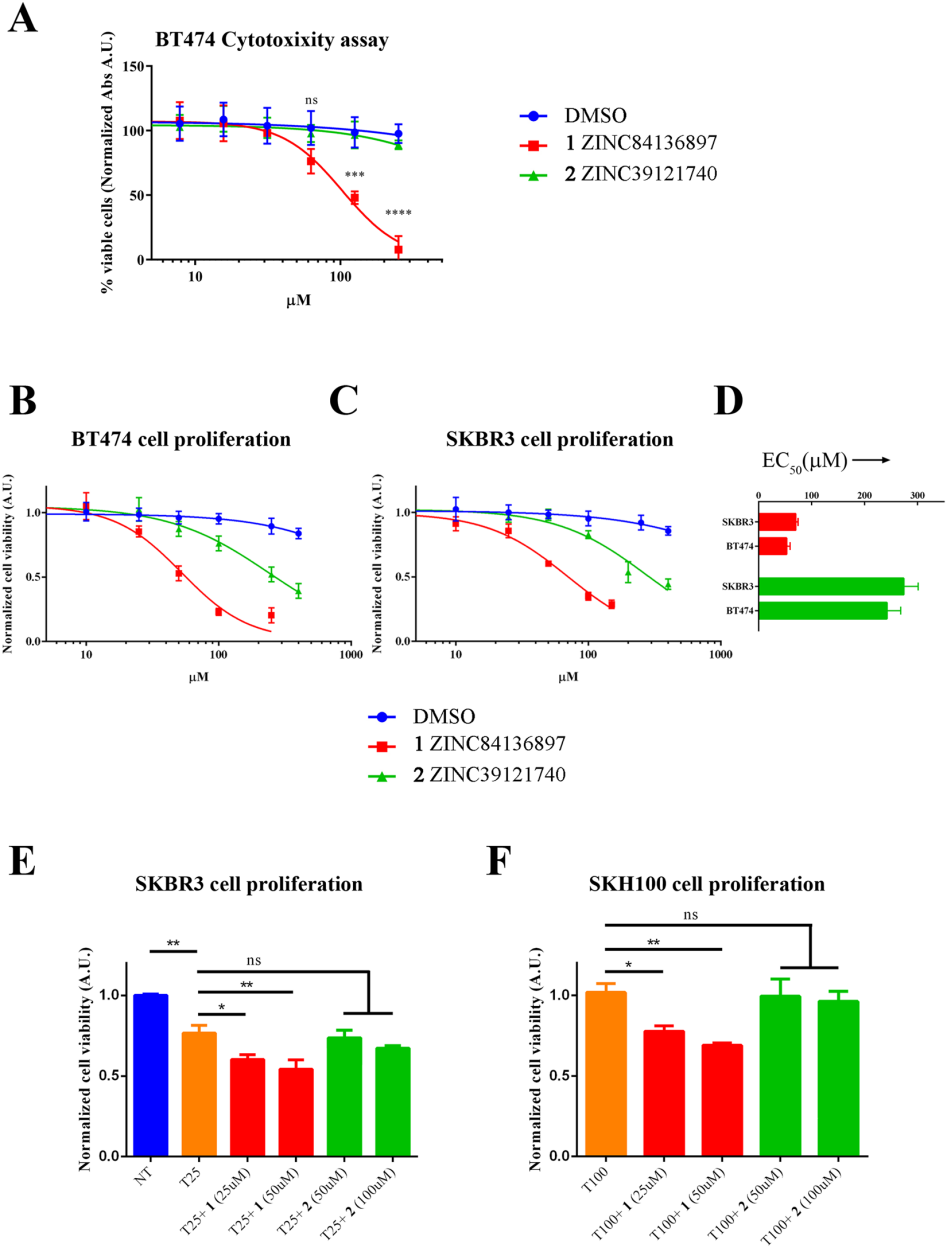
Impact of **1** and **2** compounds on BT474 breast cancer cells. (**A**) To assess whether **1** and **2** could trigger cytotoxic effects on living cells, BT474 cells were treated with serial dilutions of **1** and **2** (or sterile DMSO). After three days, live cells were detected by standard MTT assay, highlighting the appearance of cytotoxic effects above 75 μM for compound **1**. Compound **2** did not show cytotoxic effects even at 250 μM. ***p < 0.001; ****p < 0.0001. (**B**) **1** and **2** compounds inhibit cell proliferation of BT474 and (**C**) SKBR3 breast cancer cell lines. Cells were treated with different concentration of the **1** and **2** and proliferation was assayed three days later by performing MTT assay. Results were fitted with four-parameter dose-response curve using GraphPad Prism7 software. (**D**) EC50 values for **1** and **2** in BT474 and SKBR3 breast cancer cell lines are represented as histogram with 95% confidence intervals. (**E**) SKBR3 cells were treated with Trastuzumab (25 μg/ml) either alone or in combination with compound **1** or **2** at the indicated doses. After three days, live cells were detected by standard MTT assay. Data are represented as mean ± SD. *p < 0.05, **p < 0.01. (**D**) SKH100 Trastuzumab-resistant cells were treated with Trastuzumab (100 μg/ml) either alone or in combination with compound **1** or **2** at the indicated doses. After three days, live cells were detected by standard MTT assay. Data are represented as mean ± SD. *p < 0.05, **p < 0.01.

*In vitro* data indicate that compounds **1** and **2** can interfere with p130Cas/ErbB2 interaction, although with different binding modalities and different affinities. To test which are the consequences of such interference in a cellular context we performed cell proliferation and cytotoxicity assays. To this end, BT474 and SKBR3 breast cancer cells were chosen as a suitable experimental model as they express high endogenous levels of both ErbB2 and p130Cas^5^. As shown in Fig. 6A, treatment of BT474 cells with serial dilutions of the compounds indicates that compound **1** exhibits toxic effects starting from 75 μM, while for compound **2** we did not detect cytotoxic effects even at 250 μM. In addition, since we already demonstrated that p130Cas/ErbB2 association triggers proliferation of breast cancer cells (as demonstrated by soft agar assay in^10^), a cell proliferation assay was employed to determine EC_50_ values of **1** and **2** compounds in BT474 (**1** EC_50_ = 52.3 ± 6.38 μM, **2** EC_50_ = 239.5 ± 25.98 μM) and in SKBR3 (**1** EC_50_ = 69,8 ± 4.44 μM, **2** EC_50_ = 273.9 ± 27.18 μM) cells as shown in Fig. 6B,C. It should be noted that both compounds achieved 50% inhibition of proliferation at concentrations below their cytotoxic threshold.

Notably, compound **1** seems to be more effective on the inhibition of cell proliferation in these specific cellular models (Fig. 6B,C). Interestingly, these compounds have no significant effect on the proliferation of ErbB2-negative HEK 293T cells (Fig. S5), suggesting that the anti-proliferative effect of **1** and **2** in BT474 and SKBR3 cells is mainly linked to the inhibition of p130Cas/ErbB2 association, rather than a broad, non-specific inhibition of p130Cas-related pathways.

Moreover, results shown in Fig. 6E indicate that compound **1** significantly enhances the efficacy of Trastuzumab treatment in SKBR3 cell line, thus suggesting that inhibition of p130Cas-ErbB2 interaction could improve Trastuzumab efficacy by decreasing the stability of ErbB2. Consistently, the treatment of SKBR3 Trastuzumab-resistant cells with compound **1** partially restores the sensitivity of resistant cells to Trastuzumab (Fig. 6F). Our data suggest that the inhibition of ErbB2-p130Cas interaction might impact on both breast cancer cell proliferation and resistance to Trastuzumab, opening up new therapeutic perspectives.

### ADME-Tox prediction

To explore the future as a drug of **1** and **2** we used free online tools to predict both physico-chemical (pK_a_ and log D^7.4^) and ADME-Tox properties. Data are shown in Table 2. According to pK_a_ prediction, both compounds are expected to be in their neutral state at physiological pH and have a similar lipophilicity. Although the ADME-Tox profile is globally acceptable, both molecules have defaults. In particular, **2** is predicted to be poorly permeable (using pkCSM predictor the threshold to distinguish highly from poorly permeable compounds is 0.90), whereas **1** is expected to be a substrate of P-glycoprotein which could adversely affect the drug candidate effectiveness.

**Table 2:** Predicted physico-chemical and ADME-Tox properties of the two investigated compounds.

	Source	1	2
pK_a_	MoKa	3.44 (b); 9.97 (a)	9.45 (a)
log D^7.4^	MoKa	1.5	2.0
TPSA	ZINC	96	72
permeability	pkCSM	1.07 × 10^−6^ cm/s	0.69 × 10^−6^ cm/s
P-glycoprotein substrate	pkCSM	Yes (inhibitor)	No
Cyp3A4 substrate	pkCSM	Yes	No
hERG toxicity	pkCSM	No	No
AMES toxicity	pkCSM	No	No

## Discussion

Despite the enormous interest in targeting Protein-Protein Interactions (PPIs), the discovery of drugs capable to interfere with these interactions has been proven to be very challenging. The transient nature of these interactions, moderate affinity, and promiscuity of recognition are among the many factors that have contributed to difficulty in discovering effective modulators^26–28^. Target validation is an essential step in the drug discovery process and one of the major causes of attrition. Therefore, the first aim of this study consisted in providing strong support for using p130Cas/ErbB2 as a target for the discovery of anticancer drugs.

We reported here, for the first time, that p130Cas association to ErbB2 receptor is indeed direct. *In silico* simulations, *in vitro* data and experiments in 293T cell line support that the direct binding is mediated by the SH3 domain of p130Cas that recognizes a polyproline type II motif located within the unstructured carboxy-terminal tail of the receptor. MD simulations suggest that this polyproline sequence is energetically favored in the interaction acting as a ligand for class I SH3 domain. This evidence partially contrasts with data reported in 1995 by Polte and Hanks^29^ showing that the direct binding of SH3 domain of p130Cas with Focal Adhesion Kinase occurs through the Pro-rich sequence –APPKPSR- that represents a class II SH3 domain ligand. However, several data reported that some SH3 domains (i. e. Src SH3 domain) can bind both class I and class II polyproline peptides^30^.

In accordance with our results, mutagenesis experiments of key amino acids in the PPII motifs allowed us to establish that among the three important residues, the cationic Arg2 residue plays a minor role in the specificity of the interaction with the SH3 domain of p130Cas. This led us to hypothesize that the essential “core” for the association of polyproline type II peptides with the SH3 domain of p130Cas is the PxxP motif, regardless of the position of the charged residue. This in turn might explain why p130Cas can associate through its SH3 domain with class I and class II peptides.

The experimental evidence of the direct interaction between p130Cas and ErbB2 is however not sufficient to establish whether this protein-protein interaction (PPI) is druggable (i.e. if it could be disrupted by a third molecule) or not.

To investigate the druggability of p130Cas/ErbB2 interaction, we firstly performed structure based virtual screening using a dataset of small molecules (SMs). This choice is in line with a recent PDB-wide analysis which shows that nearly half of all PPIs may be susceptible to SM inhibition^31^.

Among chemicals predicted to be valuable inhibitors of the investigated interaction we submitted to experimental validation two compounds exhibiting a different binding mode: **1** exploits its action by interacting with p130Cas in three regions, whereas the interaction of **2** with p130Cas is driven by hydrogen bonding (HB) interactions.

*In vitro* experiments showed that the two compounds similarly inhibit PPI and suggest that molecules with different binding modes could share similar inhibition properties. Conversely, cell line experiments showed that **1** may stronger slow down cell proliferation in BT474 and SKBR3 cell line (models of ErbB2-overexpressing breast tumor) than **2**. Permeability prediction could explain this behavior since **1** is expected to be significantly more permeable than **2**.

We previously demonstrated that high levels of p130Cas expression inversely correlate with ErbB2 sensitivity to Trastuzumab probably by binding to ErbB2 and increasing its stability to the cell membrane^5^.

Interestingly, a small molecule inhibitor (compound **1**) able to interfere with p130Cas/ErbB2 association synergized with Trastuzumab treatment in Trastuzumab-sensitive SKBR3 cells, thus suggesting that inhibition of p130Cas-ErbB2 interaction might be used to improve Trastuzumab efficacy. Notably, this beneficial effect was also observed in Trastuzumab-resistant cells, in which compound **1** partially restores their sensitivity to Trastuzumab. These effects might rely on the ability of compound **1** to interfere with p130Cas/ErbB2 association, thus reducing p130Cas-mediated ErbB2 stabilization at the cell membrane^5^. These results strongly enforce the hypothesis that the increased stabilization of ErbB2 by p130Cas might be a crucial event driving breast cancer progression and resistance to antibody-based anti-ErbB2 therapies, opening new therapeutic alternatives.

In this regard, a medicinal chemistry effort is required to optimize compounds’ potency. To this end, virtual libraries of analogues of **1** and **2** will be obtained, screened for their activity using structure-based virtual screening (SBVS) as above, and then filtered based on synthetic accessibility and physicochemical properties. These procedures allow to test the most promising candidates in order to find an optimized lead compound.

Overall, this study provides a potential hit compound that through a number of optimization steps could gain relevance as an anticancer drug candidate and proposes a strategy to discover small molecules which may target protein-protein interactions. It is worth noting that the results reported in this study represent the first attempt to translate the current large knowledge on p130Cas, and its contribution to breast cancer progression, into something that may be employed as therapeutic agent in the future.

## Experimental Section

### Cloning of p130Cas

Coding sequence for p130Cas SH3 domain (3–71) (named SH3_p130Cas) was amplified from cDNA with PCR using 5′-GGATCCCACCTGAACGTGCTGGC-3′ forward primer and 5′-GCGGCCGCCTATTATGGCTTCTTATCATACA-3′ reverse primer and cloned in-frame into pGEX-4T3 vector using BamHI and NotI restriction sites.

pGEX vectors containing p130Cas substrate domain (named YXXP_**p130Cas**) and p130Cas carboxy-terminal domain (named CT_**p130Cas**) were engineered by Prof. Emilia Turco, MBC, University of Torino. All PCR amplifications and cloning procedures were verified by Sanger sequencing.

### Cloning of ErbB2

Two different ErbB2 constructs containing the putative polyproline domain (named shortPPII_**ErbB2** and longPPII_**ErbB2**) were amplified from ErbB2 cDNA and cloned in-frame in pMAL-C2 vector for recombinant protein production and in pEGFP-C2 vector for 293T transfection, using SacI and SalI restriction sites.

### Expression and purification of recombinant proteins

pGEX-4T3 and pMAL-C2 engineered vectors were purified using commercial silica column and transformed into competent BL21 bacteria for expression of recombinant fusion proteins. *MBP* and *GST* fusion proteins were produced in *Escherichia coli* BL21 bacteria strain together with *GST* and *MBP* proteins alone (*GST*_ctrl and *MBP*_ctrl, respectively) as controls and then purified on a Sepharose amylose (New England Biolabs) or Sepharose glutathione column (GE Healthcare) according to the manufacturer’s instructions. After elution step all recombinant proteins were dialyzed two times against PBS at 4 °C, aliquoted and stored at −80 °C. See Table 1 for recombinant proteins details.

### Site directed mutagenesis

Site directed mutagenesis of shortPPII_**ErbB2** sequence (in pMAL-C2 and pEGFP-C2 vectors) was performed with QuickChange Site-Directed Mutagenesis Kit (Agilent) following manufacturer instructions. Mutagenic primers were the same for both plasmids (Table S1).

Mutants of shortPPII_**ErbB2** protein in pMAL-C2 vector were expressed, purified and quantified as reported above. See Table 1 for recombinant protein details.

Site directed mutagenesis of SH3_**p130Cas** sequence (in pGEX-4T3 vector) was performed with QuickChange Site-Directed Mutagenesis Kit (Agilent) following manufacturer instructions. Mutagenic primers were reported (Table S1). Mutants of SH3_**p130Cas** protein in pGEX-4T3 vector were expressed, purified and quantified as reported above.

### Antibodies

Mouse monoclonal antibodies to human p130Cas were produced in our department (see^5^) Additional mouse monoclonal p130Cas antibodies were purchased from BD Transduction Laboratory (Franklin Lakes, NJ, USA) (Material number 610272). Antibodies to *GST* (mouse monoclonal), *MBP* (rabbit polyclonal) and *GFP* (mouse monoclonal and rabbit polyclonal) tags were produced at the MBC, University of Torino. Additional rabbit polyclonal antibodies to *GST*, *MBP* and *GFP* tags were obtained from Thermo Scientific (catalog # A-5800) and Sigma Aldrich (catalog #M1321 and # G1544).

### Dot blot binding assay

For dot blot experiments, 1 μl of *GST*-tagged p130Cas purified recombinant proteins was manually spotted onto nitrocellulose and let air-dry for 30 minutes at room temperature. Saturation was performed using 5% BSA in TBS-0.3% Tween20 at room temperature for 3 hours. After 3 washing steps with TBS-0.3% Tween20, nitrocellulose filters were incubated with *MBP*-tagged ErbB2 purified recombinant proteins (1 μg/ml) in Interaction Buffer (50 mM Tris-HCl pH7.4, 150 mM NaCl, 1%Triton-X100) at 4 °C overnight. The day after, nitrocellulose filters were washed twice with Interaction Buffer and twice with TBS-0.3% Tween20. The interacting proteins were detected using rabbit polyclonal anti-*MBP* antibody and peroxidase conjugated anti-rabbit secondary antibody with chemoluminescent ECL reagent.

Nitrocellulose filters were then extensively washed with TBS-0.3% Tween20 and incubated overnight at 4 °C with mouse monoclonal anti-*GST* antibody and detected by peroxidase conjugated anti-mouse secondary antibody. This process revealed the amount of *GST*-tagged p130Cas purified recombinant proteins that were directly spotted onto nitrocellulose.

### *In vitro* binding assay

For the *in vitro* binding assay, *GST*-tagged p130Cas and *MBP*-tagged ErbB2 purified recombinant proteins were mixed together in solution (Interaction Buffer with 1% BSA) overnight at 4 °C. The day after, standard immunoprecipitation with anti-*MBP* rabbit polyclonal antibody and proteinA Sepharose (GE Healthcare) was performed. After 3 washing steps, proteins were eluted with 2X Laemmli loading buffer. Proteins were run on SDS-PAGE under reducing condition, transferred to nitrocellulose and probed with specific antibodies. Interacting partners were revealed using anti-*GST* mouse monoclonal antibody.

### ELISA

For ELISA binding assay, 100 nM/well of *GST*_SH3_**p130Cas** and *GST*_ctrl purified recombinant proteins were coated to a 96-well microtiter plate in 100 μl of PBS buffer overnight at 4 °C. The day after, washing steps and saturation were performed using PBS and 3%BSA in PBS at room temperature. *MBP*-tagged ErbB2 purified recombinant proteins and *MBP*_ctrl (100 nM/well) were added in 100 μl/well of PBS for 2 h. After washing, anti-*MBP* mouse monoclonal antibody (0.25 μg/ml) was added for 2 h followed by peroxidase conjugated anti-mouse secondary antibody for 1 h. Detection of the protein-protein interaction was revealed by standard colorimetric reaction adding 90 μl of chromogenic TMB substrate (3,3′,5,5′-tetramethylbenzidine). The reaction was stopped with 90 μl of 0.5 M HCl and absorbance at 450 nM was measured using ELISA plate reader. *GST*_ctrl and *MBP*_ctrl proteins served as control for non-specific binding between recombinant proteins.

For validation of inhibitory activity of selected compounds, ELISA binding assay was setup as reported above. Briefly, **1** and **2** compounds were mixed at various concentration with *MBP*_shortPPII_**ErbB2** purified recombinant protein and allowed to interact with pre-absorbed *GST*_SH3_**p130Cas** 2 h at room temperature. Detection of the protein-protein interaction was revealed as reported above. DPN (Diarylpropionitrile, from Sigma Aldrich, catalog number H5915, CAS Number 1428-67-7) was used as control for non-specific inhibition of protein-protein interaction^32^.

### Inhibitory compounds

ZINC84136897 (**1**) and ZINC39121740 (**2**) were purchased from Enamine Ltd. (Kiev, UA) as lyophilized powder and dissolved at 25 mM concentration in 75% DMSO/water. Solubilized chemicals were aliquoted and stored at −20 °C protected from light.

Standard MS and H^1^NMR were performed to validate structure and quality.

### Cell cultures

HEK 293T cells (from ATCC, CRL3216) were maintained in DMEM (Thermo Fisher Scientific), 10% FBS at 37 °C, 5% CO2 in humidified incubator.

BT474 cells (from ATCC, HTB20) were cultured in DMEM-F12 (Thermo Fisher Scientific), 10% FBS at 37 °C, 5% CO2 in humidified incubator.

SKBR3 cells were cultured in McCoy’s 5 A (Thermo Fisher Scientific), 15% FBS at 37 °C, 5% CO2 in humidified incubator.

SKBR3 resistant cells (SKH100) made resistant to Trastuzumab were generated by Dr. Valabrega and maintained as described in^33^.

All used cell lines were authenticated in the last 6 months by BMR Genomics (Padova, Italy), using the CELL ID System (Promega, Madison, WI).

All cells were regularly checked for mycoplasma contamination by specific PCR.

### 293T transfection

For transfection of pEGFP-C2 vectors containing *GFP*-tagged ErbB2 constructs, 293T cells were seeded in 10-cm dishes at 5 × 10^6^ cells/dish. After cells reached 90–95% confluence, plasmid vectors (8 μg/dish) with Lipofectamine 2000 (Invitrogen Inc.) were transfected into 293T cells following manufacturer instructions.

### Cytotoxicity assay

For cytotoxicity assay BT474 cells were seeded at 5000 cells/well concentration (100% confluency) in a 96-well cell culture plate and incubated with serial dilutions of **1** and **2** compounds (from 250 μM to 0.12 μM). Cytotoxicity was evaluated after 3 days by MTT assay (11465007001 Roche, Cell Proliferation Kit I) following manufacturer instruction.

### Cell proliferation assay

For proliferation assay BT474, SKBR3 and SKH100 cells were seeded in 8 replicates at 1000 cells/well concentration (25% confluency) in four different 96-well cell culture plates and then incubated with increasing concentration of **1** and **2** for 3 days. Changes in proliferation rate were detected by MTT assay (11465007001 Roche, Cell Proliferation Kit I) following manufacturer instruction. Sterile DMSO was used as control for non-specific solvent effect on proliferation.

### Statistical analysis

All the results are representative of at least three independent experiments performed in triplicate.

All data obtained on protein-protein interaction were normalized using *GST*_ctrl and *MBP*_ctrl as non-specific control. Data obtained from ELISA and MTT assays using **1** and **2** were normalized using either DPN or DMSO as non-specific control.

Statistical analysis of data obtained from ELISA and MTT assays was performed using 2-way ANOVA corrected for multiple comparison by Tukey test. p-value < 0.05 (95% confidence interval) were considered significant.

### Fluorescence quenching experiments

All fluorescence spectra were recorded with a Horiba Jobin Yvon Fluorolog3 TCSPC spectrofluorophotometer with 1.0 cm quartz cells

For fluorescence experiments, *GST*_SH3_**p130Cas** was firstly tagged with fluorescein (Fl-*GST*_SH3_**p130Cas**) using a methodology described in the literature^34^ see Supporting Information). The fluorescence spectra of Fl-*GST*_SH3_**p130Cas** were obtained using a fixed concentration of 3.0 μM in the absence and in the presence of *MBP*_shortPPII_**ErbB2** (concentration range: 0.05–3.0 μM). Fluorescence emission spectra of Fl-*GST*_SH3_**p130Cas** in the presence of **1** and **2** were obtained using the following concentrations: Fl-*GST*_SH3_**p130Cas** = 0.7 μM; **1** and **2**  = 0–21 μM. Finally, *MBP*_shortPPII_**ErbB2** (concentration range: 0–75 μM) was added to the complexes between Fl-*GST*_SH3_**p130Cas** and the two compounds.

The variation in fluorescence emission at 518 nm of Fl-*GST*_SH3_**p130Cas** was monitored in all experiments.

### Computational section

In this study, we used two crystallographic structures: the SH3 domain of p130Cas (PDB code 1WYX) and the SH3 domain of p85 in complex with the peptide PD1R (PDB code 3I5R). Both structures were downloaded from the Protein Data Bank (www.rcsb.org).The ErbB2 sequence was submitted to Eukaryotic Linear Motif (ELM) (www.elm.org)^18^ by using the FASTA format as an input.

The interaction complex SH3_p130Cas/PPII_**ErbB2** was built with MOE version 2010.10 (www.chemcomp.com).

The virtual screening for inhibitors of the SH3_p130Cas/PPII_**ErbB2** interaction has been performed by FLAP version 2.0.2 (www.moldiscovery.com). The screening has been made over the 15 million compound subset “drug-like clean” of the ZINC database v.13 (www.zinc.docking.com).

### Preparation of the complex SH3_p130Cas/PPII_ErbB2

Two starting structures were downloaded from the Protein Data Bank (www.rcsb.org): the SH3 domain of p130Cas (PDB code 1WYX) and the SH3 domain of p85 in complex with the peptide PD1R (PDB code 3I5R). 1WYX and 3I5R were imported in MOE (ver. 2016.08, www.chemcomp.com).

Firstly, PD1R 3D structure was extracted from 3I5R. Four amino acids, i.e. K, L, L and S, present in the PD1R sequence (**K**RP**L**PP**L**P**S**) were mutated into V, Q, S and R, respectively, to obtain PPII_**ErbB2** model (**V**RP**Q**PP**S**P**R**).

The structure of the SH3 domain of p130Cas was prepared starting from 1WYX: one monomer was retained, and water molecules and ions were removed. The structure was then protonated using MOE Protonate3D (T = 310,15 K, pH = 6,8, I.F. = 0,2 mM). The same procedure was applied to the SH3 domain of p85 present in the pdb file 3I5R.

Finally, the structure of the complex SH3_p130Cas/PPII_**ErbB2** was obtained through alignment and superposition of SH3_p130Cas and PPII_**ErbB2** with the SH3 domain of p85 and PD1R, respectively.

The complex was minimized using the CHARM22 force field (RMS gradient less than 0.001 kcal mol^−1^ Å^−2^).

### Molecular Dynamics simulations

All steps of MD simulations were set up using the BiKi Life Science software (default settings for plain MD simulations, ver. 1.3.5, http://www.bikitech.com/) which provides an intuitive GUI interface to GROMACS and Amber tools. In particular, the setup of the simulations is based on Antechamber software (ver. 14, http://ambermd.org/antechamber/ac.html) whereas MD simulations were performed with GROMACS (ver.4.6.1, http://www.gromacs.org/) package using the Amber ff14 force field^35^. Water molecules were described using the TIP3P model^36^ and periodic boundary conditions were applied. The system was neutralized by adding a sodium ion.

The solvated system was minimized using a steepest descent minimization (the maximum number of minimization cycles was set to 5000). Equilibration was carried out in four steps: firstly, three 100 ps NVT simulations were performed to gradually increase the temperature up to the final 300 K, then one 1 ns NPT step was carried out to allow the system to stably reach the pressure condition of 1 atm. Finally, one 50 ns MD production run was performed with a time step for integration equal to 0.002 ps and a number of steps equal to 25000000. Coordinates were saved every 10 ps; in total 5000 snapshots were obtained. The temperature coupling was done using a velocity rescaling with a stochastic term that ensures that a proper canonical ensemble is generated^37^.

### Dataset preparation

The subset of compounds named “Clean Drug-Like” was downloaded from the ZINC database (ver. 13, www.zinc.docking.org). This subset contains about 15 million of molecules which were filtered by applying the following criteria: (a) Lipinski’s rules of five (i.e. no more than 5 hydrogen bond donors and 10 hydrogen bond acceptors, a molecular mass less than 500 Daltons, ClogP less than 5), (b) molecular mass greater than 150 Daltons, (c) number of rotatable bonds lower than 7, (d) formal charge in the range [−2; +2].

### Structure based virtual screening (SBVS)

The SH3_**p130Cas**/PPII_**ErbB2** structure was imported in FLAP (v. 2.2.1, www.moldiscovery.com) and the interface between the protein and the peptide was used to define the pocket for the SBVS (Pocket Point Radius = 2 Å). MIFs with the default probes DRY, O, N1, and H were calculated for the residues in the pocket and FLAP fingerprints for SH3_p130Cas were generated^38^.

The compounds resulting from the previous step were submitted to FLAP in their ionization state at pH = 7. FLAP generates 25 low energy conformers for each compound. For each of these conformations, the molecular interaction fields (MIFs) for H, O, N1, and DRY GRID probes were calculated at a 0.75 Å grid resolution, and FLAP fingerprints were generated. To reduce the number of molecules in the dataset, a quick pre-filtering run was performed using the implemented procedure in the bit-string mode. The Glob-Sum similarity score was used to rank the compounds in the dataset and the 10000 compounds with the higher score were retained.

Finally, the reduced dataset was screened against the SH3_p130Cas model scoring the complementarity between the candidate and the protein pocket. The Glob-Prod similarity was used to rank the molecules in the dataset.

### Physico-chemical and ADME-Tox prediction

For calculating pK_a_ and log D^7.4^ we used MoKa v. 2.6.5 (www.moldiscovery.com). To predict the pharmacokinetic profile of **1** and **2** we used pkCSM (http://biosig.unimelb.edu.au/pkcsm/), a free Web tool, properly documented in the literature^39^.

All calculations were run on an Aethia EXA-W 4 core Xeon workstation.

## Supplementary information

Supplementary information
