# Author Correction: Modeling ErbB2-p130Cas interaction to design new potential anticancer agents

**DOI:** 10.1038/s41598-019-54252-5

**Published:** 2019-11-22

**Authors:** Andrea Costamagna, Matteo Rossi Sebastiano, Dora Natalini, Matilde Simoni, Giorgio Valabrega, Paola Defilippi, Sonja Visentin, Giuseppe Ermondi, Emilia Turco, Giulia Caron, Sara Cabodi

**Affiliations:** 1 Department of Molecular Biotechnology and Health Sciences, University of Torino, Via Nizza 52, 10126 Torino, Italy; 2 Institut für Pharmacologie, Universität Bern, Inelspital INO-F, CH-3010 Bern, Switzerland; 3 Candiolo Cancer Institute, FPO - IRCCS -, Candiolo, (TO) 10060 Italy

Correction to: *Scientific Reports* 10.1038/s41598-019-39510-w, published online 28 February 2019

In the original version of this Article, Giorgio Valabrega was incorrectly affiliated with ‘Department of Medical Sciences, University of Torino, Torino, Italy’. The correct affiliation is listed below.

Candiolo Cancer Institute, FPO - IRCCS - Candiolo (TO) 10060, ITALY

This error has now been corrected in the PDF and HTML versions of the Article.

