# Supplementary material for: Modeling ErbB2-p130Cas interaction to design new potential anticancer agents
Source: Sci Rep. 2019 Feb 28;9:3089. doi: 10.1038/s41598-019-39510-w (PMC6395809; doi:10.1038/s41598-019-39510-w)
Supplement: Supplementary file 1 — Supplementary information [file 41598_2019_39510_MOESM1_ESM.pdf]

SUPPLEMENTARY INFORMATION

For

Modeling ErbB2-p130Cas interaction to design new potential anticancer agents

Andrea Costamagna[1§], Matteo Rossi Sebastiano[2§], Dora Natalini[1], Matilde Simoni[1], Giorgio Valabrega[3], Paola Defilippi[1], Sonja Visentin[1], Giuseppe Ermondi[1], Emilia Turco[1], Giulia Caron[1*], Sara Cabodi[1*]

Table of contents

Figure S1. SH3_p130Cas/PPII_ErbB2 Molecular Dynamics: negative control

Figure S2. SH3_p130Cas/PPII_ErbB2 Molecular Dynamics: binding orientation

Figure S3. GST-tagged recombinant proteins for p130Cas domains.

Figure S4. Analysis of equilibrium dissociation constants and fitting

Figure S5, S6, S7. Uncropped full-length blots of Figure 2 and Figure 3

Table S1. List of primers for site directed mutagenesis of PPII_ErbB2

Table S2. SBVS results: top ranked structures

Supplementary Methods:

Bioconjugation experiments

Dye/Protein ratio calculation

Fluorescence data analysis

Molecular dynamics simulations to clarify the binding orientation of the SH3 domain to the polyproline domain

PDB Coordinates

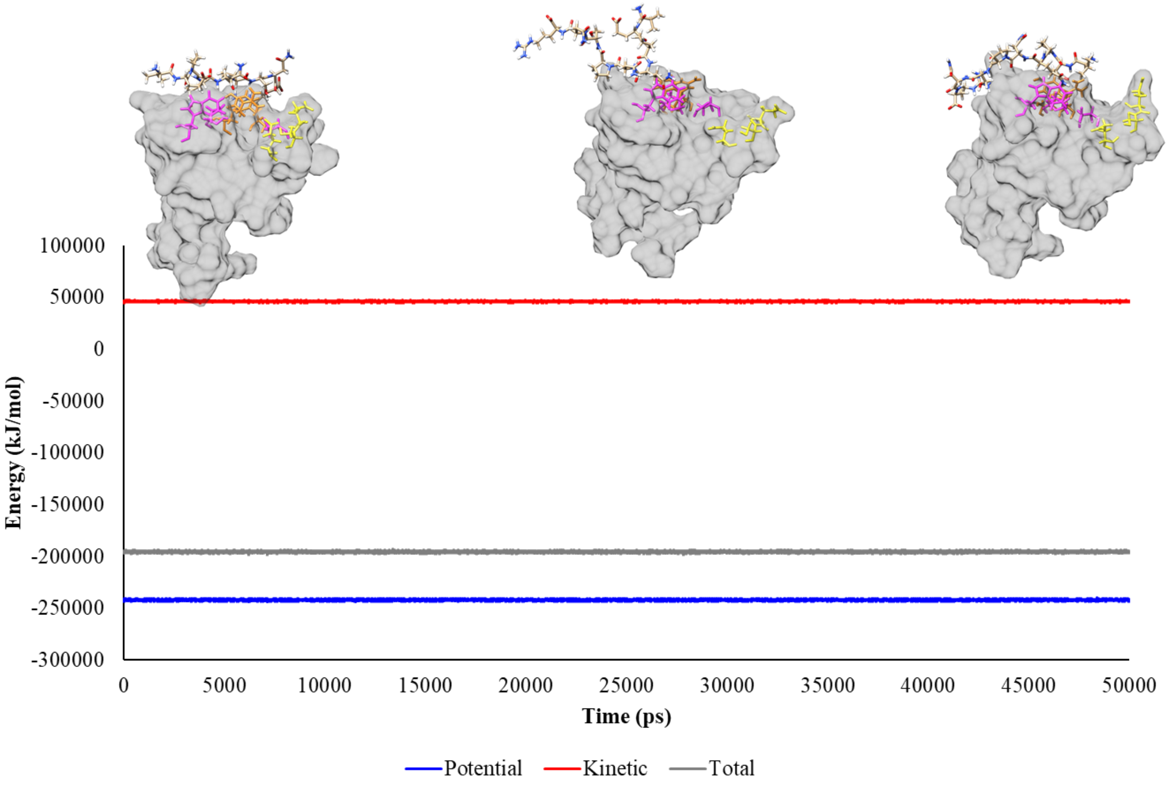

Figure S1. SH3_p130Cas/PPII_ErbB2 Molecular Dynamics: negative control

As a negative control we mutated the three PPII residues mainly involved in the interaction into amino acids less prone to stabilize the binding (positive charged Arg2 into negative charged Glu, small Pro5 and Pro8 into the larger Asn) and applied the same MD protocol. Results show a weaker interaction between the mutated peptide and SH3_p130Cas. The energy variation with the time is shown together with three snapshots of the trajectory.

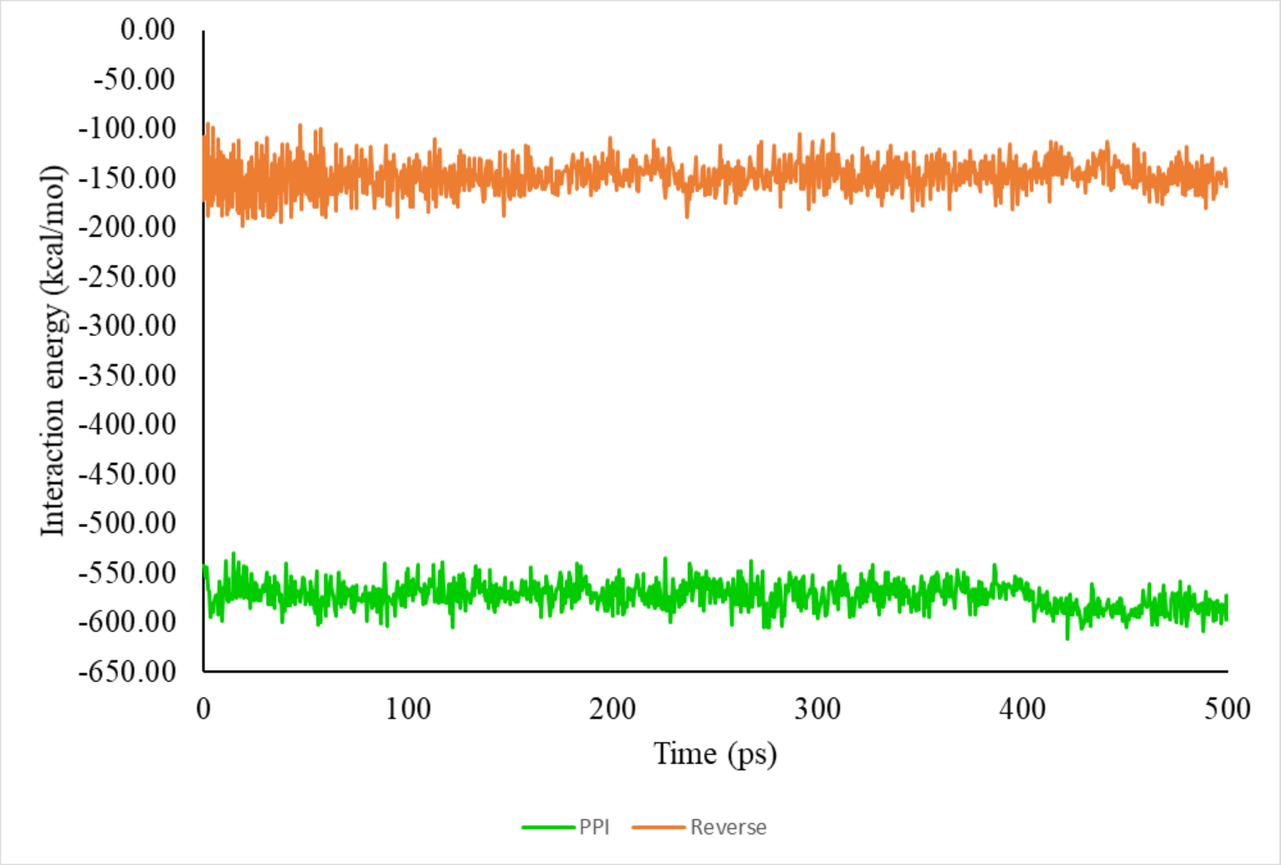

Figure S2. SH3_p130Cas/PPII_ErbB2 Molecular Dynamics: binding orientation

MD simulations were also successfully used to support the binding orientation, – VRPQPPSPR – of the SH3 domain to the polyproline domain. The interaction energy variation is showed with the time for the two complexes SH3_p130Cas/PPII_ErbB2 (green) and SH3_p130Cas/reverse PPII_ErbB2 (orange).

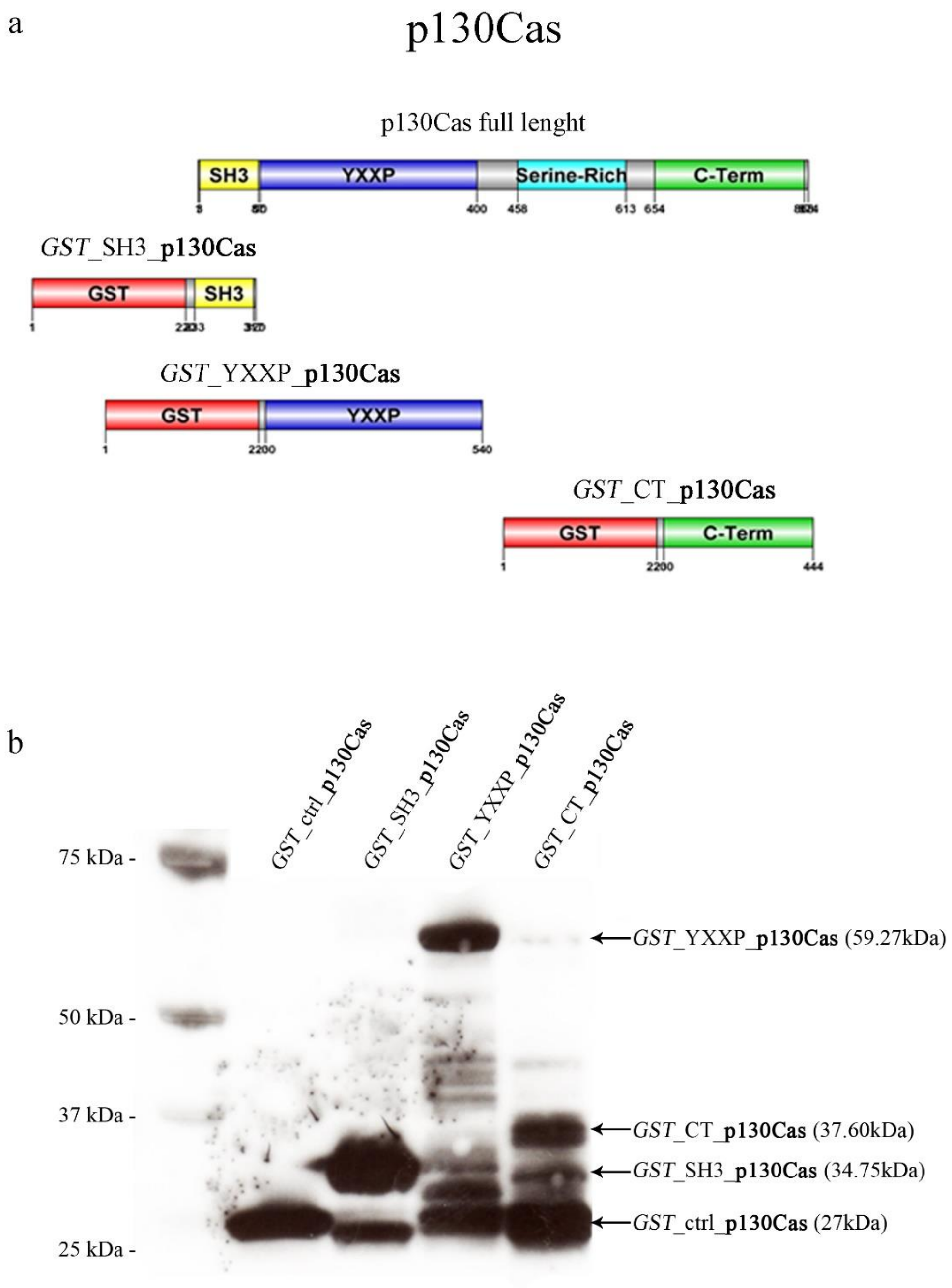

Figure S3: GST-tagged recombinant proteins for p130Cas domains.

a) Schematic representation and b) western blot for p130Cas recombinant proteins. See Table 1 in the main text for additional information.

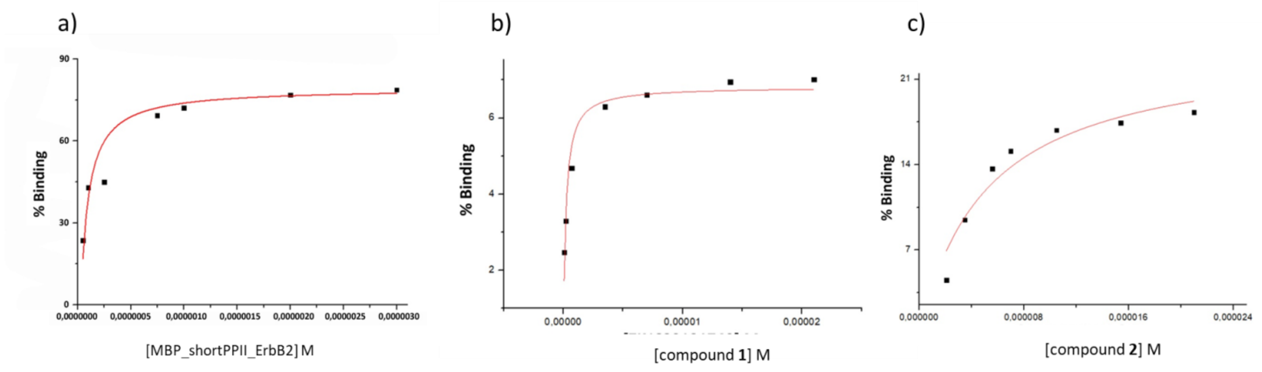

Figure S4. Analysis of equilibrium dissociation constants and fitting.

Data analysis of sample by non linear fit: a) Fl-GST_SH3_**p130Cas**/ MBP_shortPPII_**ErbB2**; b) Fl-GST_SH3_**p130Cas**/**1; c)** Fl-GST_SH3_**p130Cas**/**2** (Pontremoli et al. 2015).

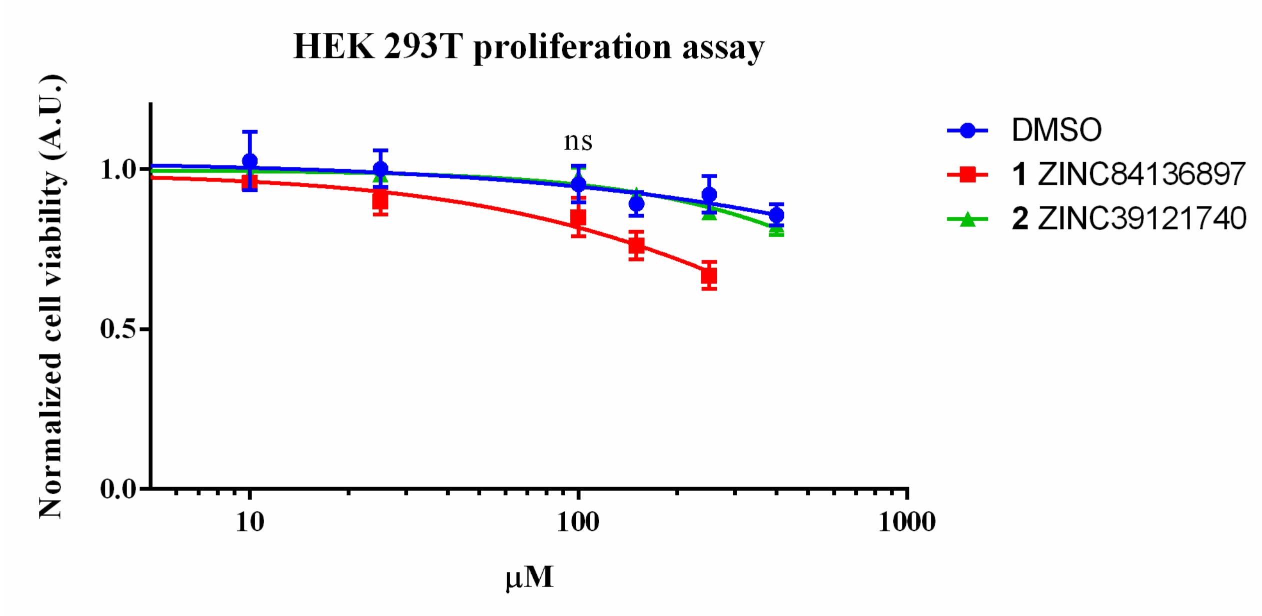

Figure S5. Proliferation assay of ErbB2-negative HEK 293T cell line treated with inhibitory compounds.

To assess whether **1** and **2** could affect cell proliferation independently from p130Cas/ErbB2 inhibition, ErbB2 ErbB2-negative cell line, HEK 293T cells were treated with serial dilutions of **1** and **2** (or sterile DMSO). After three days, live cells were detected by standard MTT assay. Compound **1** had no significant effect on cell proliferation at concentrations that were indeed effective in BT474 and SKBR3 cell lines (see Figure 6B and 6C); while compound **2** did not have any effect even at high concentration.

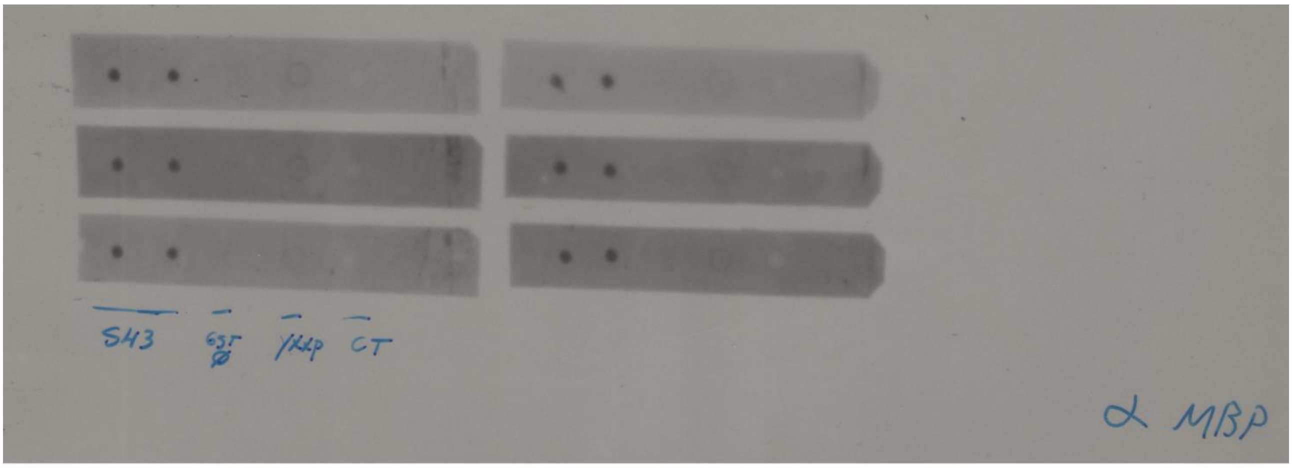

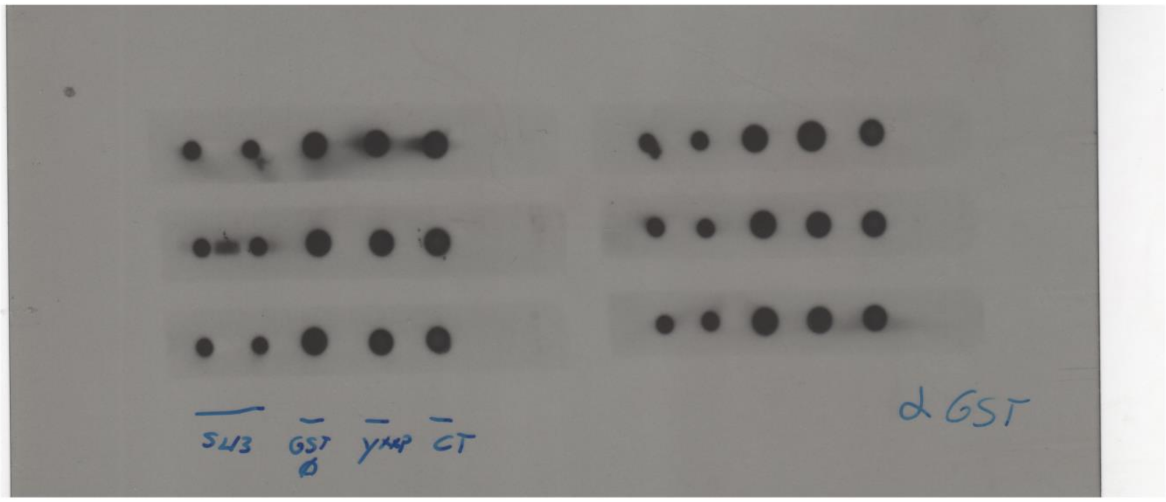

Figure S6. Uncropped blots for dot blot experiment showed in Fig. 2.

Multiple replicates were performed to minimize technical artifacts introduced by the manual spotting of recombinant proteins onto nitrocellulose membrane.

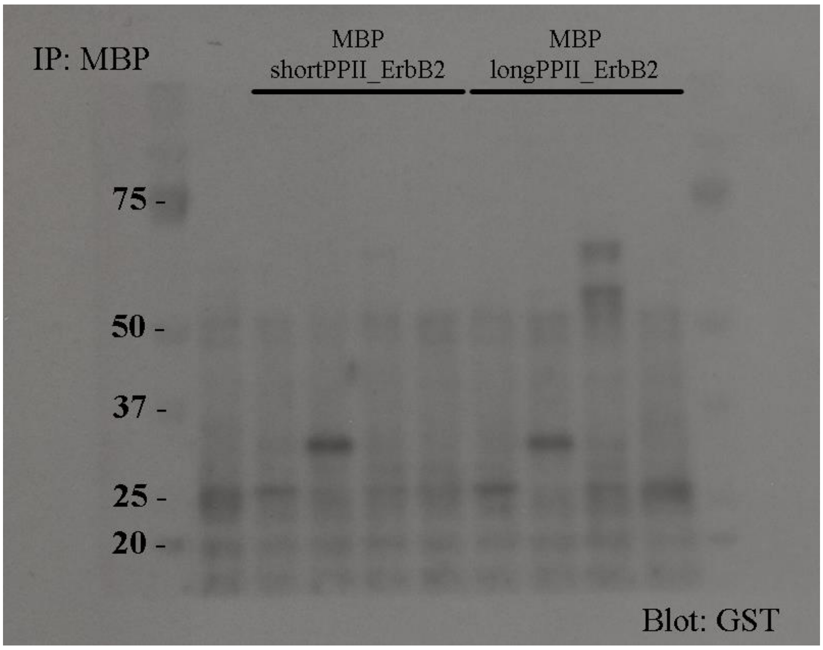

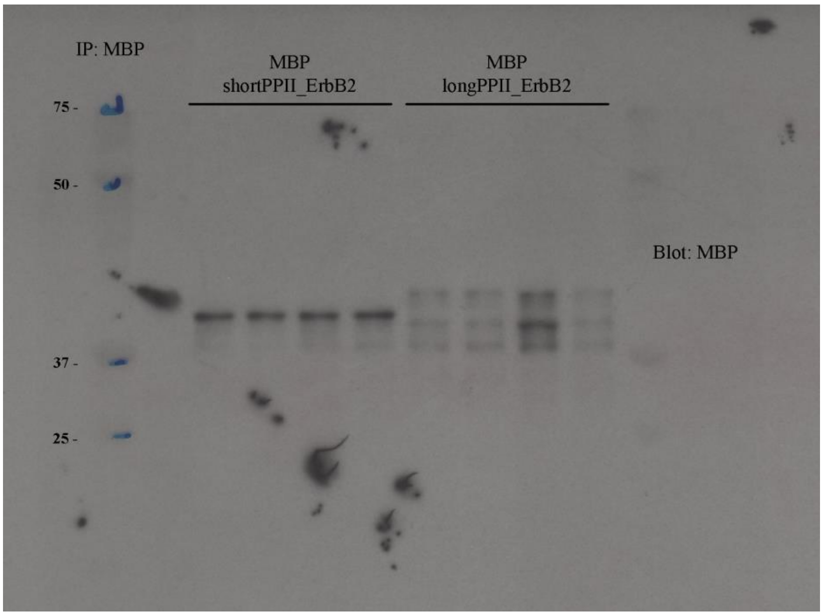

Figure S7. Uncropped full-length blots for in vitro binding experiment showed in Fig. 2.

To better differentiate between molecular weight of the various recombinant proteins each immunoprecipitation was splitted and loaded in two precast protein gels with different polyacrilamide percentage. The first lane on the left after the MW marker is the result of the immunoprecipitation with non-specific antibodies.

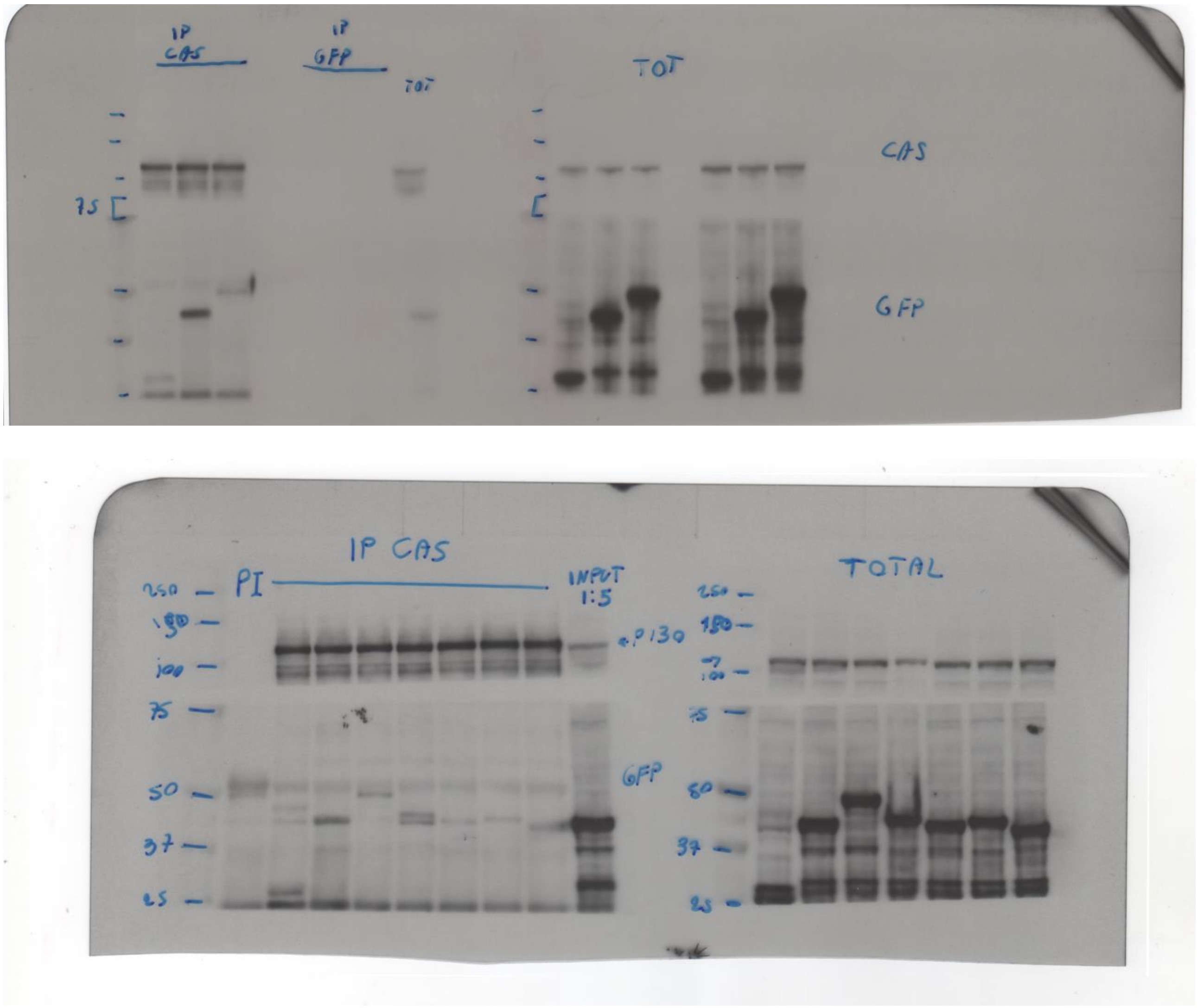

Figure S8. Uncropped full-length blots for immunoprecipitation experiments showed in Fig. 3. In the first image, it should be noted that our anti-GFP antibody could not suitable for immunoprecipitation. In this blot total cell lysates from transfected 293T cells were loaded in different amount and run twice (upper figure, right panel).

In the lower image, the first lane on the left after the MW marker is the result of the immunoprecipitation with non-specific antibodies. All the GFP-tagged variants of ErbB2 were expressed at similar levels when transfected in HEK 293T cells (lower image, right panel).

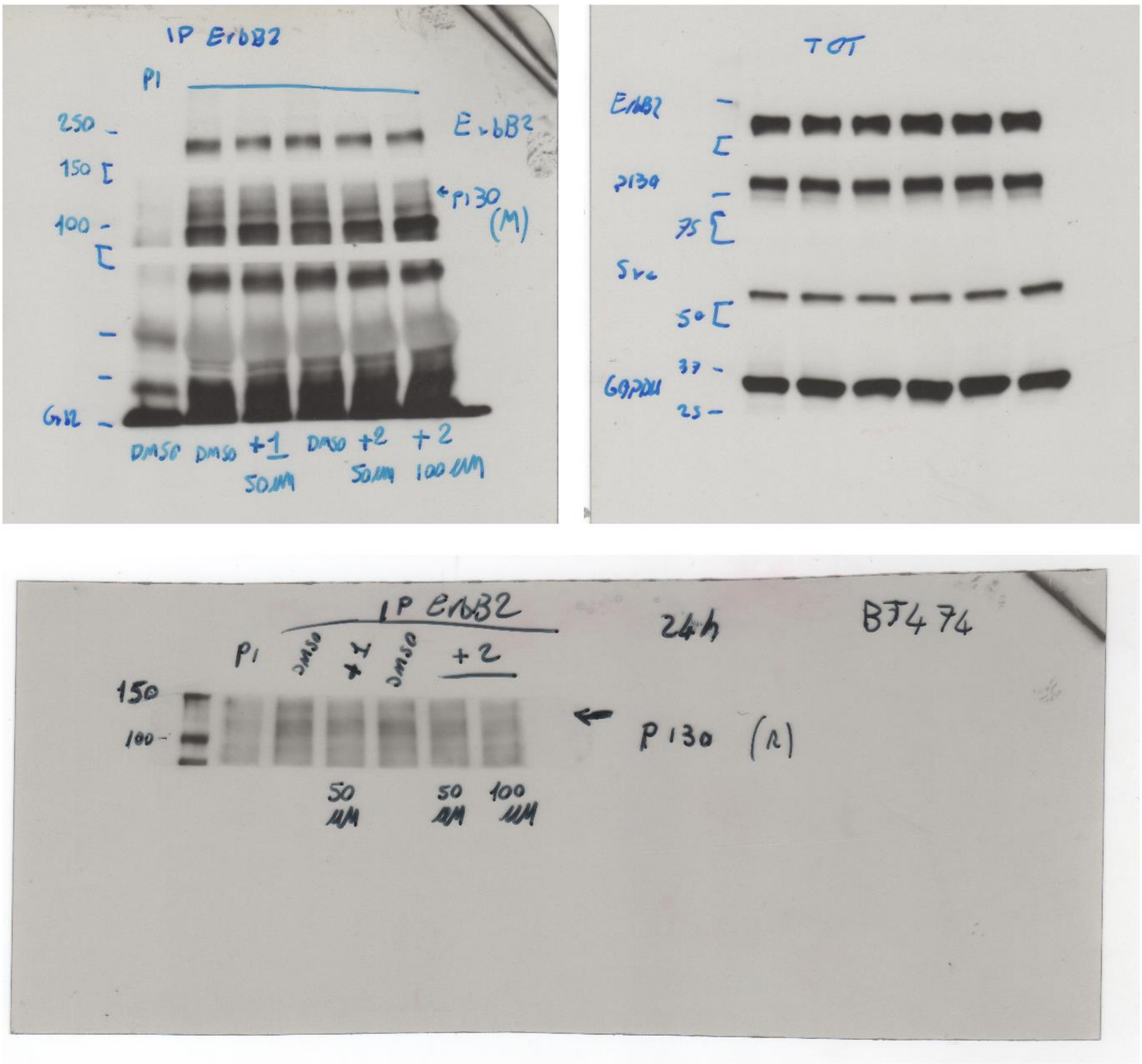

Figure S9. Uncropped full-length blots for immunoprecipitation experiments showed in Fig. 4. Co-immunoprecipitation of p130Cas was probed with two different antibodies raised in different species (mouse monoclonal in the upper panel, rabbit polyclonal in the lower panel).

shortPPII_ErbB2	FW	RW
R → A	AAGGGGGCTGGGGATCAACATCTGGCTGGTTCACATATTCA	TGAATATGTGAACCAGCCAGATGTTGATCCCCAGCCCCCTT
PP → AA	CTCTCGGGCCGAAGGGGCCTGGGGCC	GGCCCCAGGCCCCTTCGGCCCGAGAG
RPP → DAA	CTCTCGGGCCGAAGGGGCCTGGGGATCAACATCTGGCTGGTT	AACCAGCCAGATGTTGATCCCCAGGCCCCTTCGGCCCGAGAG
deleter	GAACCAGCCAGATGTTCGAGAGGGCCCTCT	AGAGGGCCCTCTCGAACATCTGGCTGGTTC
SH3_p130Cas		
Y10A	GATTCAGCAACATTGTCAGCGAGGGCTTTGGCCAGCAC	GTGCTGGCCAAAGCCCTCGCTGACAATGTTGCTGAATC
E15W	AGCTCATCCGGGGACCAAGCAACATTGTCATAGAGGGCTTTGGC	GCCAAAGCCCTCTATGACAATGTTGCTTGGTCCCCGGATGAGCT
W41A	TGAGCAGAGCCACGCGCCATCCAGGCCC	GGGCCTGGATGGCGCGTGGCTCTGCTCA

Table S1. List of primers for site directed mutagenesis of PPII_ErbB2 and SH3_p130Cas

Ranking	Candidate	Glob-Prod	Interaction sites
1	ZINC69340852	0.781161	1, 2, 3
**2**	**ZINC39121740**	**0.765370**	**3**
**3**	**ZINC84136897**	**0.765167**	**1, 2, 3**
4	ZINC55361134	0.765112	3
5	ZINC94544502	0.764802	3
6	ZINC94546825	0.762961	3
7	ZINC94546804	0.762942	2, 3
8	ZINC30693240	0.762457	3
9	ZINC94546805	0.760678	2, 3
10	ZINC00844852	0.760202	3

Table S2. SBVS results: top ranked structures.

Top ranked structures from SBVS in decreasing order of Glob-Prod value. The number of interaction sites is also shown. Tested compounds **1** (ZINC84136897) and **2** (ZINC39121740) are showed in bold. The total number of screened compounds after filtering (see text for details) is 8500000, the rank values are between 0.000000 and 0.781161.

SUPPLEMENTARY METHODS

Bioconjugation experiments

GST protein has been bioconjugated with fluorescein-5- maleimide following the reported protocol[1]. The sulfhydrylcontaining protein GST was dissolved at a concentration of 1–10 mg/ml in 20 mM sodium phosphate, 0.15 M NaCl, pH 7.2. Fluorescein-5-maleimide was then dissolved in DMF at a concentration of 10 mM protecting it from light and a 25-fold molar excess of fluorescein-5-maleimide solution was added to the protein solution. After for 4 hours of reaction at room temperature in the dark the crude derivative was immediately purified using gel filtration on PD10 columns Sephadex G-25 (Amersham Bioscience) using a phosphate buffer saline solution (10 mM PBS, pH 7.4) as eluent. The solutions were protected from light during the chromatography.

Dye/Protein ratio calculation

The dye/protein ratios (D/P) of the conjugates were determined by the absorption spectra of the labelled proteins, registered in 10 mM PBS (pH 7.4) according to the relationship reported in Equation 1 [2]:

$$\mathrm{D/P} = \frac{A_{\mathrm{max}} \cdot \varepsilon_{\mathrm{prot}}}{(A_{280} - cA_{\mathrm{max}}) \cdot \varepsilon_{\mathrm{dye}}}$$

where A280 is the absorption of the conjugate at 280 nm; Amax is the absorption of the conjugate at the absorption maximum of the corresponding fluorescein-5-maleimide; c is a correction factor which must be used to adjust for amount of A280 contributed by the dye because fluorescent dyes also absorb at 280 nm and equals the A280 of the dye divided by the Amax of the dye (c = 0.29); $\varepsilon_{protein}$ (55310 cm^{-1} M^{-1}) and ε_{dye} (63096 cm^{-1} M^{-1}) are the molar absorption coefficients for the protein used and fluorescein-5- maleimide, respectively. The bioconjugation was repeated several times and the resulting D/P ratio obtained was around 1 with values varying from 0.94 to 1.6.

Fluorescence data analysis

The obtained spectroscopic data were analysed by a last equation used to analyse the experimental data is non linear least-squares fit procedures reported in Equation 2 [2]:

$$y = \frac{B_{max}\,[Q]}{K_d + [Q]}$$

in which y is the binding derived by measuring fluorescence intensity, [Q] is the concentration of tested compound, B_{max} is the maximum amount of protein/compound complex (Fl-*GST*_SH3_**p130Cas**/compound) formed at saturation and K_d is the equilibrium dissociation constant. The percentage of bound Fl-*GST*_SH3_**p130Cas**, that is, y, derived from the fluorescence intensity emission maximum, is plotted against the concentration of the tested compounds (**1** and **2**). The binding curves are reported in Fig. S4.

Molecular dynamics simulations to clarify the binding orientation of the SH3 domain to the polyproline domain.

The identified polyproline sequence in ErbB2 protein – VRPQPPSPR – representing a ligand for class I SH3 domain (N to C orientation), could be read as a palindromic class II SH3 ligand - RPSPPQPRV – in a C to N orientation (reverse).

Two complexes were investigated: the complex with PPII_**ErbB2** and the "reverse" peptide (the peptide was obtained from SH3_p130Cas/PPII_ErbB2 model by mutating the sequence of PPII_**ErbB2** into "RPSPPQPRV").

MD results reveal sensibly higher U_{ab} values (Figure S1) for SH3_p130Cas/reverse PPII_ErbB2 complex suggesting that the reverse sequence orientation is not energetically favored. This MD simulation therefore seems to validate – VRPQPPSPR – polyproline sequence as a ligand for class I SH3 domains.

MD was performed with MOE using a AMBER99 force field, Nosé-Poincaré-Andersen equations and generalized Born implicit solvent model at T = 300K.

The system was equilibrated for 100 ps at 300K and then a 500 ps MD production run was performed with a time step for integration equal to 0.002 ps. Coordinates were saved every 0.5 ps; in total 1000 snapshots were obtained.

To overcome problems due to the lack of explicit solvation, only SH3_p130Cas atoms in a radius of 4.5 Å from peptide atoms were submitted to MD without any constrains.

PDB Coordinates

The structure of the SH3 domain of p130Cas and p85 subunit of PI3K were downloaded from the PDB (PDB code 1WYX and 3I5R respectively).

SUPPLEMENTARY REFERENCES

1. Barbero, N. *et al.* Fluorescence anisotropy analysis of protein-antibody interaction. *Dyes and Pigments* **83**, 225-229 (2009).
2. Pontremoli, C., Barbero, N., Viscardi, G. & Visentin, S. Mucin-drugs interaction: The case of theophylline, prednisolone and cephalexin. *Bioorg Med Chem* **23**, 6581-6586 (2015).
